# A Triple High Throughput Screening for Extracellular Vesicle Inducing Agents With Immunostimulatory Activity

**DOI:** 10.3389/fphar.2022.869649

**Published:** 2022-04-11

**Authors:** Nikunj M. Shukla, Fumi Sato-Kaneko, Shiyin Yao, Minya Pu, Michael Chan, Fitzgerald S. Lao, Yukiya Sako, Tetsuya Saito, Karen Messer, Tomoko Hayashi, Howard B. Cottam, Maripat Corr, Dennis A. Carson

**Affiliations:** ^1^ Moores Cancer Center, University of California San Diego, La Jolla, CA, United States; ^2^ The Herbert Wertheim School of Public Health and Longevity, University of California San Diego, La Jolla, CA, United States; ^3^ Department of Rheumatology, Graduate School of Medical and Dental Sciences, Tokyo Medical and Dental University (TMDU), Tokyo, Japan; ^4^ Department of Medicine, University of California San Diego, La Jolla, CA, United States

**Keywords:** exosome (vesicle), NFkB, HTS, compounds, immune, extracellular, adjuvant, reporter cells

## Abstract

Extracellular vesicles (EVs) play an important role in intercellular communication and regulation of cells, especially in the immune system where EVs can participate in antigen presentation and may have adjuvant effects. We aimed to identify small molecule compounds that can increase EV release and thereby enhance the immunogenicity of vaccines. We utilized a THP-1 reporter cell line engineered to release EV-associated tetraspanin (CD63)-Turbo-luciferase to quantitatively measure EVs released in culture supernatants as a readout of a high throughput screen (HTS) of 27,895 compounds. In parallel, the cytotoxicity of the compounds was evaluated by PrestoBlue dye assay. For screening immunostimulatory potency, we performed two additional independent HTS on the same compound library using NF-κB and interferon-stimulated response element THP-1 reporter cell lines. Hit compounds were then identified in each of the 3 HTS’s, using a “Top X″ and a Gaussian Mixture Model approach to rule out false positive compounds and to increase the sensitivity of the hit selection. Thus, 644 compounds were selected as hits which were further evaluated for induction of IL-12 in murine bone-marrow derived dendritic cells (mBMDCs) and for effects of cell viability. The resulting 130 hits were then assessed from a medicinal chemistry perspective to remove compounds with functional group liabilities. Finally, 80 compounds were evaluated as vaccine adjuvants *in vivo* using ovalbumin as a model antigen. We analyzed 18 compounds with adjuvant activity for their ability to induce the expression of co-stimulatory molecules on mBMDCs. The full complement of data was then used to cluster the compounds into 4 distinct biological activity profiles. These compounds were also evaluated for quantitation of EV release and spider plot overlays were generated to compare the activity profiles of compounds within each cluster. This tiered screening process identified two compounds that belong to the 4-thieno-2-thiopyrimidine scaffold with identical screening profiles supporting data reproducibility and validating the overall screening process. Correlation patterns in the adjuvanticity data suggested a role for CD63 and NF-κB pathways in potentiating antigen-specific antibody production. Thus, our three independent cell-based HTS campaigns led to identification of immunostimulatory compounds that release EVs and have adjuvant activity.

## Introduction

Although vaccination against common pathogens is gaining broader acceptance, there remains an unmet need for widely effective adjuvants that can elicit sustained immune responses to targeted antigens (Reed et al., 2013). Vaccine adjuvants act as immunopotentiators that are co-administered with subunit, inactivated or attenuated antigens (Tregoning et al., 2018). In the past decade, there have been advances with adjuvants that have improved the response to varicella, influenza and hepatitis B vaccines in populations with reduced immune responses (Tregoning et al., 2018). Although the adjuvants boost protective efficacy of the vaccines, they often elicit local inflammation at the site of injection in some cases accompanied by flu-like symptoms, reduce patient acceptance especially for vaccines that require annual or booster injections (Petrovsky, 2015; Nanishi et al., 2020). Adjuvants that utilize intracellular communication pathways to enhance antigen presentation and the needed cognate cellular interactions could potentially activate the immune system in a manner that is not as abruptly inflammatory.

**FIGURE 1:**
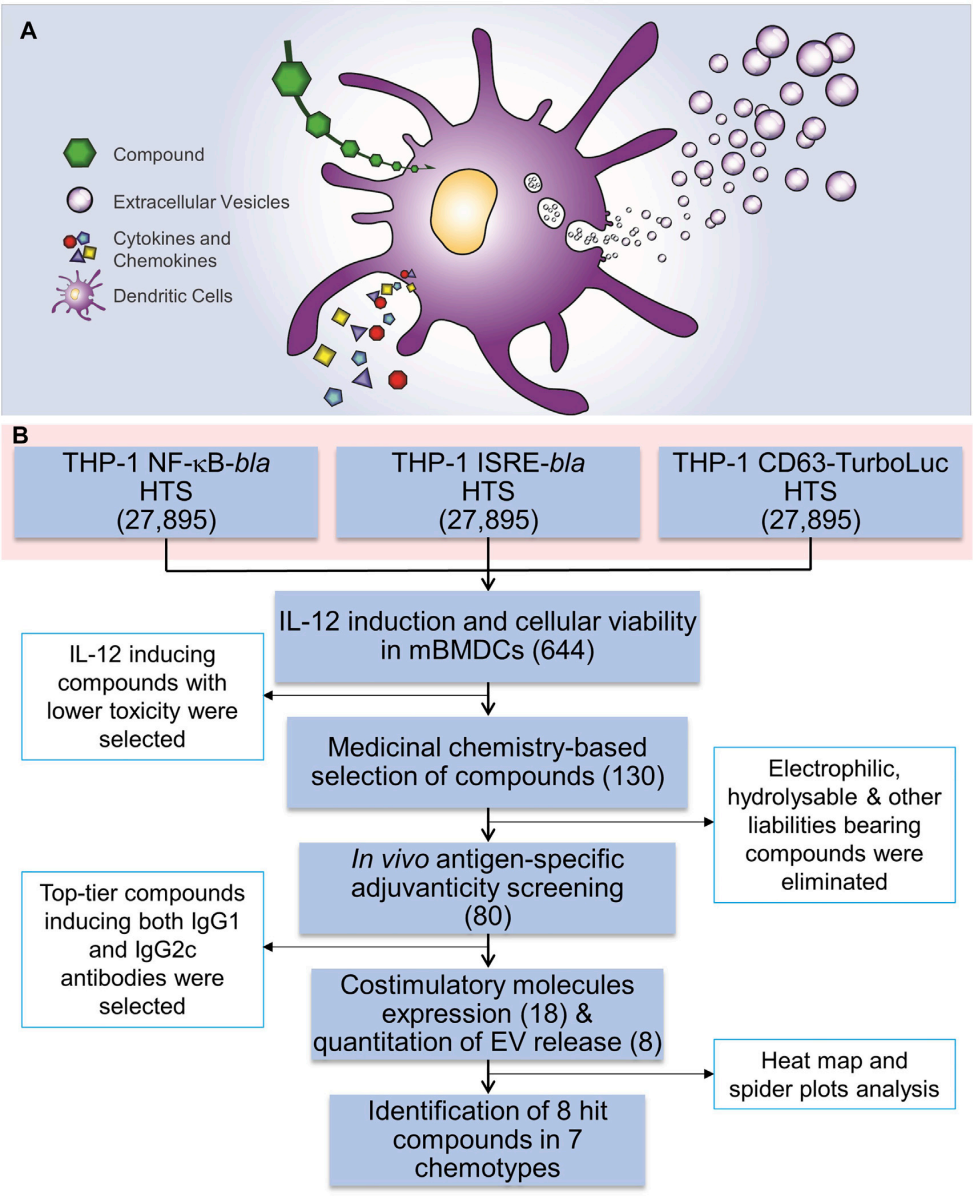
HTS for immunostimulatory compounds that enhance EV release and the HTS workflow. **(A)** A cartoon depicting the rationale for identification of compounds that enhance EV release as well as induction of cytokines and chemokines. **(B)** Three independent THP-1 cell-based high throughput screenings were performed using NF-κB-beta-lactamase (*bla*), ISRE-*bla* and CD63-Tluc-CD9-EmGFP reporter cells. These assays evaluated 27,895 compounds in duplicate and 644 compounds were identified as hits using two different statistical methods. These compounds were cherry-picked and were subjected to screening for immune stimulating activity, including induction of cytokine IL-12 and evaluation of cytotoxicity using MTT assay in mBMDCs which identified 130 compounds. Further medicinal chemistry approaches eliminated 50 compounds and the remaining 80 compounds were subjected to *in vivo* adjuvanticity screening, followed by co-stimulatory molecule expression screening and quantitation of EVs released from BMDCs. Eight distinct compounds were identified that belonged to 7 different chemotypes. The number in parentheses corresponds to the number of compounds.

Extracellular vesicles (EVs) act as a carrier of cell-type-specific molecules including those involved in innate immune responses, such as cytokines, chemokines, adhesion molecules, other proteins, lipids, peptides, coding and non-coding RNAs (including microRNAs), and DNA fragments (Valadi et al., 2007; Skog et al., 2008; Cossetti et al., 2014; Yanez-Mo et al., 2015). Adhesion molecules integrated into the EV outer surface membrane direct binding to potential target cells while other molecules act as ligands to cellular receptors. EVs can also encapsulate additional proteins or nucleic acids that can convey specific intercellular communications. These properties enable EVs to play modulating roles in mediating immune responses to pathogens and tumors (Campos et al., 2015; Wang et al., 2017). Hence, we hypothesized that small molecule compounds which can simultaneously enhance innate immune responses and EV biogenesis and release (Figure 1A) could add immunomodulation modalities, and potentially increase antigen delivery to distal lymphoid organs, leading to improved vaccine efficacy and reduced toxicity.

Recently, we developed and characterized a human monocytic leukemia THP-1 reporter cell line engineered with a fusion construct for the expression of EV-associated tetraspanins (CD63 and CD9) linked to Turbo-luciferase (Tluc) and Emerald Green Fluorescent Protein (EmGFP) (CD63 Tluc-CD9-EmGFP THP-1 cells) to quantitatively measure release of EVs in culture supernatants (Shpigelman et al., 2021). Using this reporter cell line, we described that Tluc activity levels correlated with concentrations of released EVs in the culture supernatant as measured by nanoparticle tracking (Shpigelman et al., 2021). Here, we utilized this cell line for a high throughput screening (HTS) of a library of 27,895 compounds. Additionally, we screened the same library with two additional THP-1 reporter cell lines for NF-κB and interferon-stimulated response element (ISRE) activation, respectively. Based on “Top X” and “Gaussian mixture model” (GMM) hit detection methods, 644 compounds were identified as hits. Further studies probing into the immunological properties as well as selection based on chemical structural features narrowed the selection to 80 compounds that were assessed *in vivo* for adjuvant activity. All these studies led to the identification of distinct chemotypes that display immunostimulatory effects and enhance the production of EVs.

## Results

### High Throughput Screenings

In prior work we identified compounds by HTS that induce NF-κB activation or prolong the activation of NF-κB and/or ISRE, using a library from a small molecule diversity collection (SMDC, UCSF) consisting of about 170,000 compounds (Pu et al., 2012; Chan et al., 2017a; Shukla et al., 2018). However, EV release assay was much more complex and required the use of expensive exosome-depleted media under precise incubation conditions. Thus, performing CD63 HTS in this large compound library, for which we had the NF-κB induction data (Pu et al., 2012) was difficult to achieve. We were therefore interested to obtain smaller compound libraries with extensive chemical space diversity. Thus, we selected commercial libraries developed by Maybridge (Leeds, United Kingdom) consisting of two subset libraries which are representative of the diversity of the two very different compound collections: 1) the Maybridge HitFinder library of 14,400 compounds representative of the entire Maybridge Screening Collection of over 53,000 members, and 2) the Maybridge HitCreator library of 14,000 compounds representative of the diversity of a collection of 550,000 compounds (Supplementary Table S1). Another benefit of the Maybridge library is a lack of CAS registry numbers for a large portion of compounds allowing for freedom of intellectual property. Compound purchase, transfer, acquisition, export and quality control led to elimination of 505 compounds (<2%) thus obtaining a final list of 27,895 compounds for the HTS (Supplementary Table S1).

The overall HTS workflow strategy is shown in Figure 1B. To identify compounds that induce both immune responses and EV release, three sets of screens were performed using the following reporter cell lines: NF-κB-*bla*, ISRE-*bla*, and CD63-Tluc-CD9-EmGFP THP-1 cells (Supplementary Table S2). In order to verify the feasibility of these assays, about 2,211 test compounds (8% of all library compounds) were randomly selected and assessed in a pilot screen for CD63 (Shpigelman et al., 2021), NF-κB and ISRE. Each screen was done in duplicate as two independent experiments (experiment 1 and 2) run on different days. The duplicate screening format allowed us to better understand the reproducibility of the activity response in the NF-κB and ISRE assays as we had found that these FRET based assay readouts have been historically less reproducible (Pu et al., 2012). Also, in the case of CD63 HTS, the pilot screen helped validate the assay (Shpigelman et al., 2021) and formed the basis for evaluating methods to be utilized for hit selection.

### Hit Selection Methods

**FIGURE 2:**
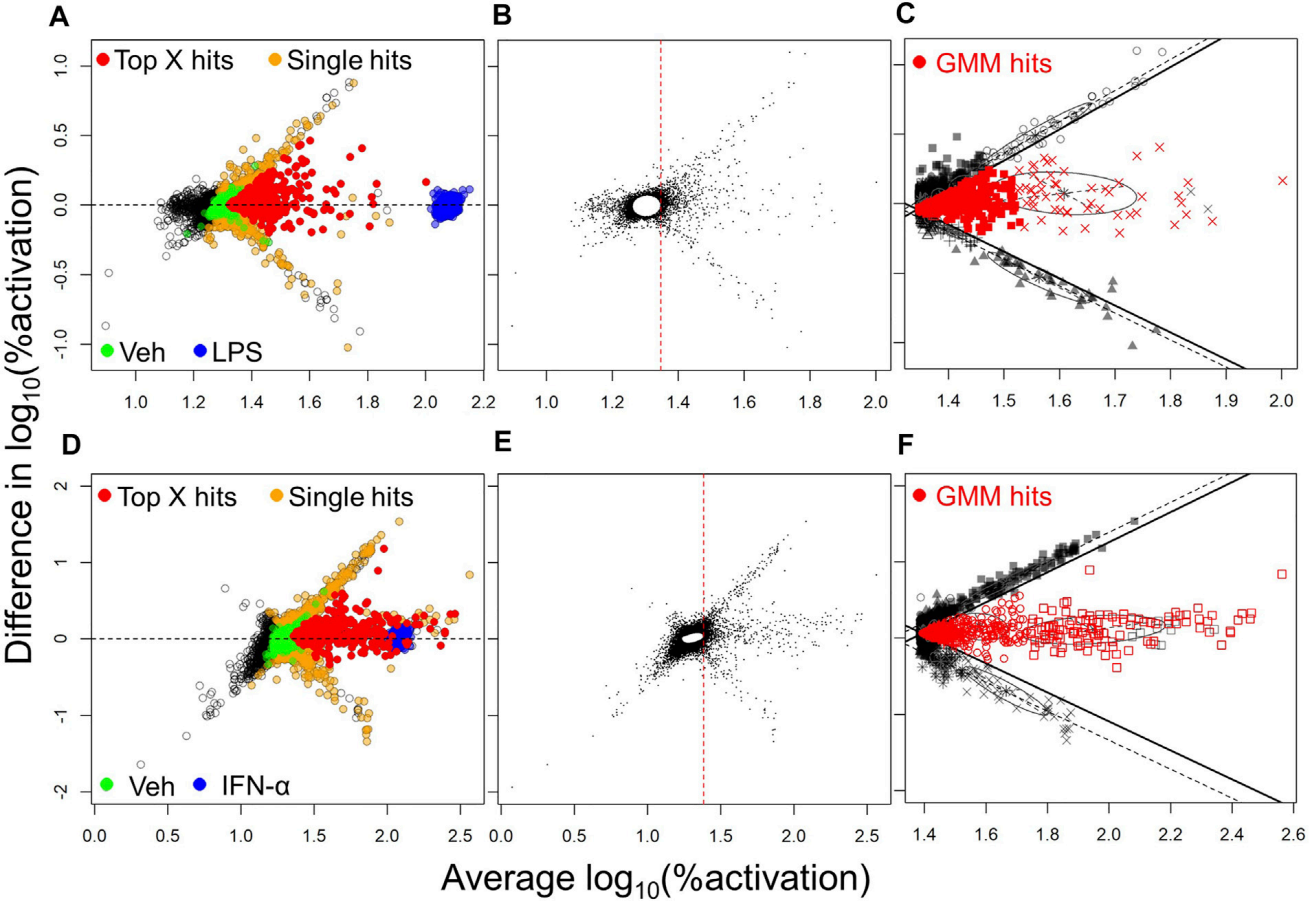
Hit selection methods from the NF-κB and ISRE HTS. The hit selection process for NF-κB HTS **(A,B,C)** and ISRE HTS **(D,E,F)** are depicted: **(A,D)** MA plots of log_10_ transformed %activation for all compounds identified as hits in one experiment (orange spheres) or in both experiments as Top X hits (red spheres). The positive (LPS for NF-κB HTS; IFN-*α* for ISRE HTS) and negative (Veh, 0.5% DMSO) controls used in the assay are shown as blue and green spheres, respectively. Panels **(B,E)** represent first steps towards the mixture model method and involved the identification of a null cluster and elimination of compounds to the left side of the red vertical dotted line. **(C,F)** The next step involved identification of linear boundaries based on the apparent false-positive clusters (black symbols) to identify GMM hits (red symbols) that included all compounds within these linear boundaries.

One of the most common methods utilized in selection of hits from HTS is Top X (McFayden et al., 2005). Thus, we initially employed this method for hit selection. Because we evaluated each compound in 2 independent experiments, we were able to eliminate many of the false positives which usually dominate FRET-based screens. To this end, an MA plot (log fold change in activation vs. average activation) was obtained by plotting the difference in the log10 %activation data for the two different experiments on the *Y*-axis, against the average of these data on *X*-axis, for each compound. These plots for NF-κB and ISRE HTS (Figures 2A,D, respectively) show the cluster of compounds (yellow circles) which were identified as hits only in one experiment and were thus considered “false positives” while the compounds that were identified as hits in both the experiments (red circles) were marked as “Top X hits.” Since we were working with a very diverse HTS library within a relatively small set of compounds, we sought to increase the sensitivity of hits detection using a Gaussian mixture modeling approach, applied separately to the NF-κB and ISRE screens. In this approach, the %activation values from the two independent experiments for each test compound were first used to construct a MA plot, and based on the plot we built a bivariate Gaussian mixture model (GMM), and this was used to cluster compounds into hit or non-hit categories. Since this method was heavily influenced by the large proportion of the non-hits, a null cluster in which the majority of compounds had activity levels similar to those of vehicle (Veh, 0.5% DMSO), was first identified (Figures 2B,E) using an initial GMM. The compounds with average %activation values lower than the maximum value of this null cluster (red dotted line, Figures 2B,E) were removed from subsequent analysis. GMM were then fitted to the remaining data, where the Bayesian Information Criterion (BIC) was used to determine the optimal number, shape and orientation of clusters. Apparent false positive clusters (grey/black colored compounds clustered along the black lines) were identified to construct linear boundaries (black lines). Compounds in the remaining clusters within these boundaries were considered to be hits (Figures 2C,F).

**FIGURE 3:**
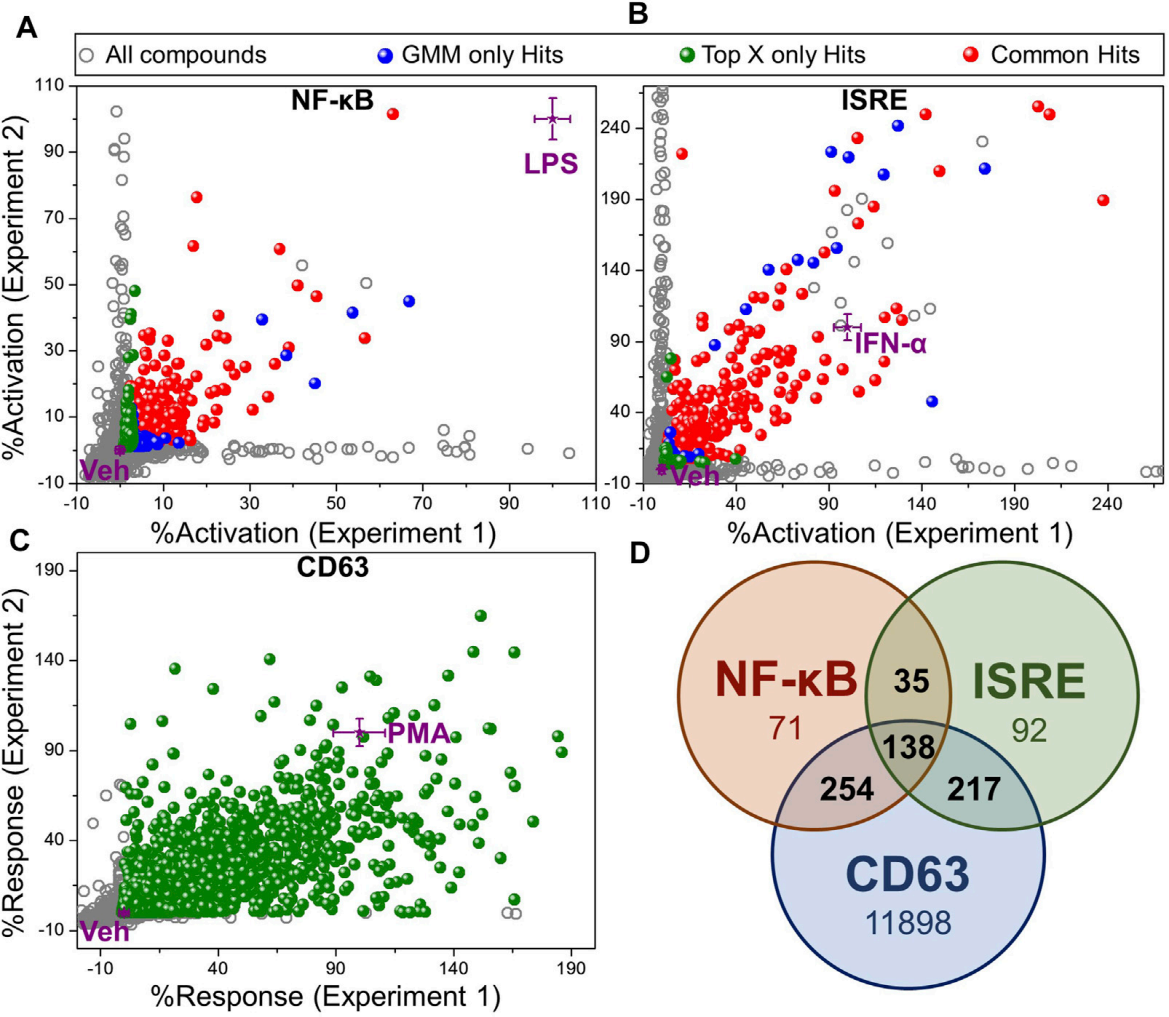
Hit selection from HTS. Scatter plot of activation data for test compounds (evaluated in duplicates) and controls from **(A)** NF-κB HTS, **(B)** ISRE HTS and, **(C)** CD63 HTS. The activation data were calculated as “%activation” based on 2-point normalization between the controls in each plate of the HTS assay. These controls included Veh (0.5% DMSO, negative control, 0%) and LPS (100 ng/ml, 100%) for NF-κB HTS, IFN-α (50 nM, 100%) for ISRE HTS and PMA (10 ng/ml, 100%) for CD63 HTS. Two different statistical methods including Top X and GMM, were utilized for hit identification. All test compounds are shown by grey dots, while the compounds that were identified as hits by Top X only or GMM only methods are shown as green and blue spheres, respectively. The compounds identified as hits by both of these statistical techniques are shown as red spheres. Controls (purple stars) are shown as mean ± standard deviation calculated by intra-assay statistics of the %activation values. **(D)** Venn diagram showing the number of compounds identified as hits in each assay after eliminating toxic compound (<40% viability) identified by PrestoBlue viability assay in CD63 HTS. Compounds confirmed as hits in at least 2 different assays as shown by numbers in the intersections of the Venn diagram were selected for the further bioactivity analysis (total 644 compounds).

Using these two methods for identification of hits, we identified 398 Top X hits and 497 GMM hits, of which 319 hits were common between both methods, as depicted by different colored spheres in Figure 3A (Supplementary Table S3). Thus, a total of 576 hits were identified from the NF-κB HTS. Similarly, for the ISRE screen, we identified 481 Top X hits and 444 GMM hits, of which 383 hits were common between both (Figure 3B) leading to a total of 542 ISRE hits (Supplementary Table S3). Figures 3A,B show the variability of %activation data between experiments 1 and 2 in both NF-κB and ISRE HTS assays, respectively.

In contrast, the CD63 HTS showed a very good correlation between the two independent experiments. Thus, we used only Top X hits, using an average of the two independent screens. To cover even weak CD63 inducers with good NF-κB or ISRE activity, we chose to use the mean of Veh wells within an assay plate as the threshold to identify Top X hits, which led to 12,954 compounds (Supplementary Table S3). To further narrow down the number of hits to compounds that have activity in a minimum of 2 out of these 3 HTS, we segregated compounds into 161 triple hits (compounds identified as a hit in CD63, NF-κB and ISRE HTS), and 3 sets of dual hits including 296 CD63 and NF-κB hits, 231 CD63 and ISRE hits and 37 NF-κB and ISRE hits (Supplementary Table S4). Then, based on the cell viability data obtained from the PrestoBlue assay, the number of hits were distributed as shown in Supplementary Table S4 with the number of hits decreasing with increasing cell viability cut-off. However, because cytotoxicity of a compound can often be circumvented by subsequent structure-activity relationship (SAR) studies, we did not want to filter out any potential hits, and thus we kept our cut-off for hit selection as compounds having more than 40% cell viability. A Venn diagram in Figure 3D shows the number of hits selected by each screen based on this 40% viability cut-off in PrestoBlue assay. Thus, a total of 644 compounds which were identified as a hit in at least 2 experiments and had viability of more than 40% by PrestoBlue were selected for further analysis.

An efficiency of compound screening methods and hit identification techniques is determined by hit confirmation rates. Thus, since all the compounds in the pilot screen were part of the HTS, we used the pilot screen as an independent confirmation screen to estimate confirmation rates. A Venn diagram was first generated for both NF-κB and ISRE screens using the number of hits from the following three sets 1. Pilot screen hits, 2. Hits identified by Top X method in HTS, and 3. Hits identified by GMM method in HTS (Supplementary Figure S1). Based on these numbers, hit confirmation rates were calculated for all three HTS (Supplementary Figure S1). Comparing the combination of Top X and clustering based hit identification methods utilized earlier for NF-κB HTS to the combination of Top X and GMM utilized here, we found increased confirmation rates from 31.5% in a prior similar HTS (Pu et al., 2012) to 67.6% in the current HTS for Top X method and 79.2% for the GMM method. The high confirmation rates were likely due to increased information available from the evaluation of compounds in duplicate. By using similar methodology, the hit confirmation rates for ISRE HTS were calculated as 57.7% for the Top X method and 60.9% for the GMM method. For CD63 HTS, the hit conformation rate was 52.6% by the Top X method only. The detailed analysis with number of hits, confirmation rates and calculations are shown in Supplementary Figure S1.

### Immunostimulatory Cytokine Induction

**FIGURE 4:**
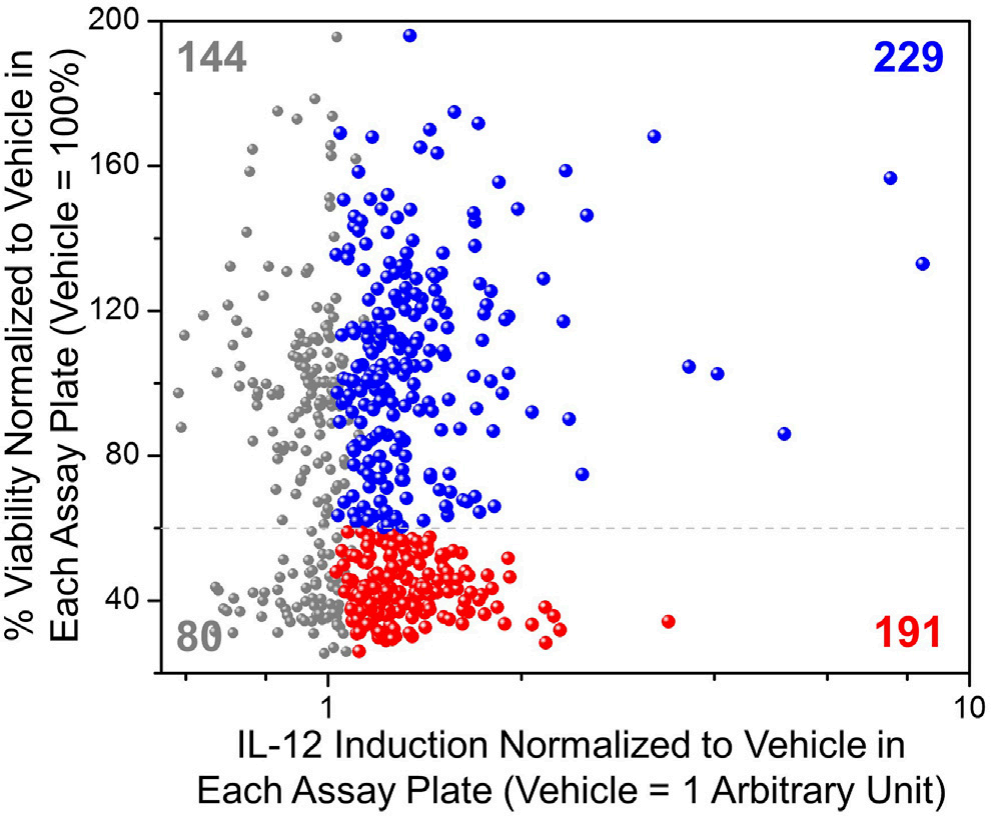
Evaluation of immunostimulatory activity and toxicity in murine BMDCs. Selected hits candidates (644 compounds) were cherry-picked and evaluated in triplicates for induction of IL-12 release and cell viability by MTT assay in mBMDCs. IL-12 induced by test compounds was normalized to IL-12 induced by Veh (0.5% DMSO, 1 Arbitrary unit) in each plate. The cell viability following treatment with each compound was normalized to the viability following treatment of cells with Veh (%viability of Veh = 100%). A scatter plot for all compounds showing normalized IL-12 induction on the *X*-axis vs. cellular viability on the *Y*-axis helped for selecting immunostimulatory compounds that were relatively less toxic. All tested compounds are shown in grey, while the compounds that induced IL-12 above mean + 3SD (standard deviation within each assay plate) of the vehicle are shown in color, of which compounds that led to cellular viabilities less than 60% are shown in red spheres (191 compounds) while relatively non-toxic (%viability ≥60%) and potent IL-12 inducing compounds are shown by blue spheres (229 compounds).

The selected 644 compounds that consisted of 138 triple hits, 254 CD63 and NF-κB hits, 217 CD63 and ISRE hits, and 35 NF-κB and ISRE hits were cherrypicked from the original HTS source plates to evaluate their immune stimulating activities in primary mBMDCs. These mBMDCs were incubated with compounds (10 μM, in triplicates) overnight and NF-κB downstream cytokine IL-12 release in the culture supernatant was measured by ELISA while the remaining cells in the plates were measured for viability by 3-(4,5-dimethylthiazol-2-yl)-2,5-diphenyltetrazolium bromide (MTT) assay. DMSO (0.5%) was used as a Veh control to determine the baseline IL-12 induction, and the IL-12 levels for all the compounds were normalized to Veh (IL-12 induced by Veh = 1). Similarly, the cell viabilities measured by MTT were normalized to Veh as 100%. The scatter plot in Figure 4 demonstrates the relative viability on the *Y*-axis and the normalized IL-12 inducing activity on the *X*-axis. We identified compounds that induced IL-12 more than 3 standard deviations above the mean of Veh in each plate and categorized them by cell viability into 2 groups. 229 compounds having viabilities above 60% (blue spheres) and 191 compounds having viabilities below 60% (red spheres), were identified as shown in Figure 4. These 229 hits were then rescreened for IL-12 induction at 5 μM compound concentration to further confirm IL-12 inducing potency which led to identification of 130 compounds that induce IL-12 more than the mean +SD above Veh in each plate (Supplementary Figure S2).

### Medicinal Chemistry Based Elimination of Hits

In an effort to narrow down our selection of hits for further *in vivo* adjuvanticity screening, we evaluated each compound structure for electrophilic characteristics, and presence of reactive and/or unstable functionalities, including Michael acceptors, hydrolyzable esters, reactive thioureas, and other indicators of pan-assay interference compounds (PAINS) (Baell and Holloway, 2010; Baell and Walters, 2014). Of the 130 compounds identified earlier, some of the compounds bearing such functionalities as shown in Supplementary Figure S3 were removed. Thus, the selected 80 compounds were sourced from the vendor and purchased in sufficient quantities (5–10 mg) to perform further bioactivity evaluation in an *in vivo* adjuvanticity screen.

### *In vivo* Adjuvanticity Screening

**FIGURE 5:**
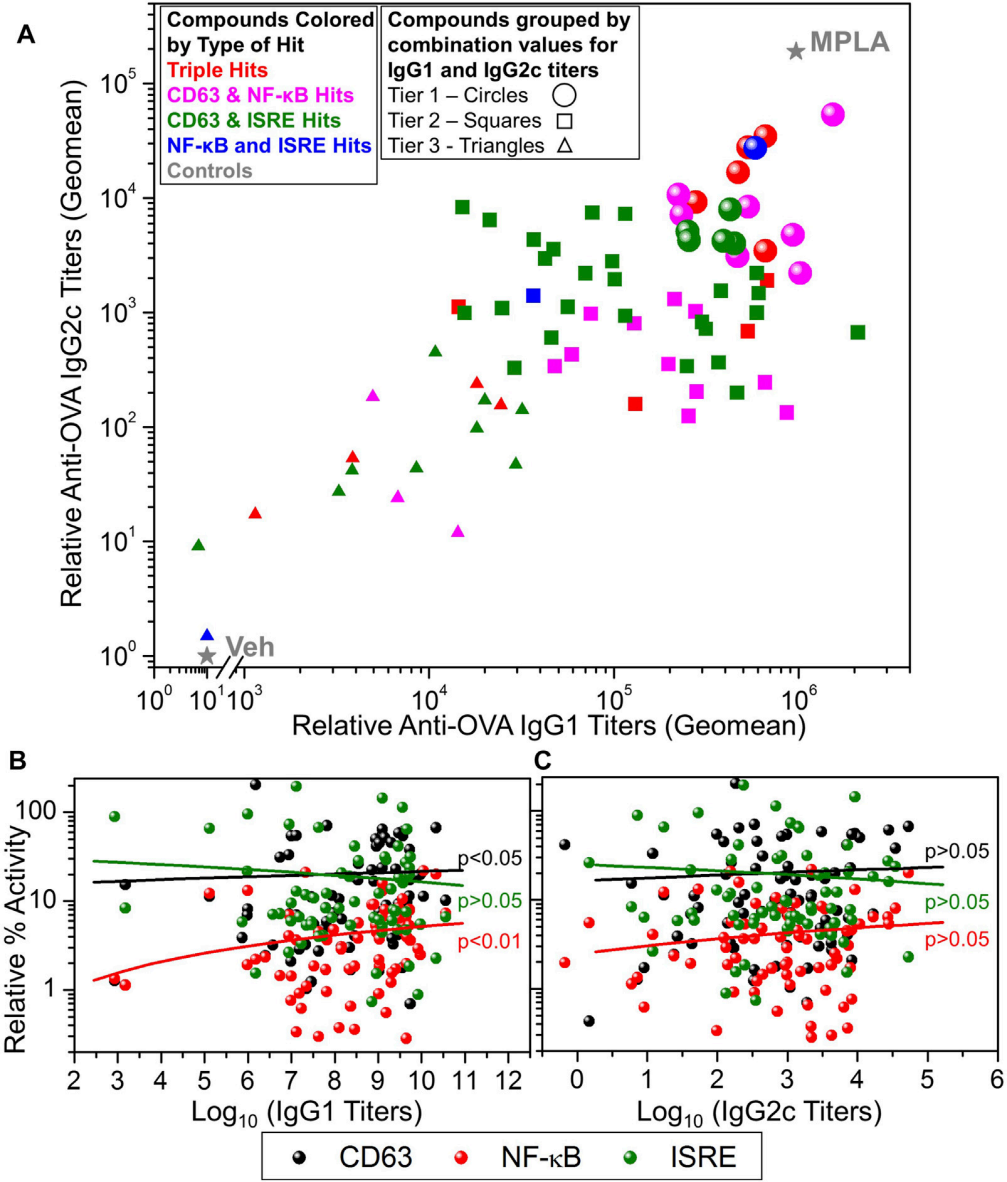
*In vivo* adjuvanticity screening of test compounds with ovalbumin as a model antigen in mice. **(A)** C57BL/6 mice (n = 3/group) were intramuscularly immunized with OVA (20 µg/mouse) as antigen and adjuvanted with test compounds (200 nmoles/mouse), or MPLA (1 µg/mouse), or Veh (10% DMSO) on days 0 and day 21 and bled on day 28 to measure OVA-specific IgG2c and IgG1 levels in sera by ELISA. A scatter plot of the geomean (n = 3) values of the IgG2c titers (*Y*-axis) and IgG1 (*X*-axis) for each compound was generated to show the adjuvant potency distribution. Compounds were represented by different symbol types based on the type of hit identified, namely, triple hits (red symbols) and dual hits including CD63 and NF-κB dual hits (magenta symbols), CD63 and ISRE dual hits (green symbols) and NF-κB and ISRE dual hits (blue symbols). MPLA was the positive control while Veh was used as the negative control shown as grey stars. Based on the potency of compound to enhance the induction of combined IgG1 and IgG2c titers, they were divided into three tiers, Tier 1 (circles), Tier 2 (squares) and Tier 3 (triangles). These tiers were obtained by first calculating log_10_ transformed values of the IgG1 and IgG2c titers and normalizing these values in between 0 and 10 (10 for MPLA and 0 for Veh). This was followed by averaging these values (for each compound) for IgG1 and IgG2c to obtain a combination value, where Tier 1 compounds had a combination value ≥8, Tier 2 compounds had a combination value ≥6 and ≤8, while Tier 3 compounds had a combination value <6. Correlation of antigen-specific IgG1 **(B)** and IgG2c **(C)** antibody titers with primary HTS (CD63, NF-κB, and ISRE) data. The data were analyzed by two-tailed nonparametric correlation (Spearman) with calculated *p*-values shown.

The selected 80 compounds were first evaluated for purity and identity by HPLC-MS and compounds which were less than 90% pure were purified by Prep-HPLC. These were then evaluated for vaccine adjuvant activity in mice using a model antigen, ovalbumin (OVA). C57BL/6 mice (n = 3 for each compound) were immunized with OVA (20 µg/animal) mixed with 200 nmol/injection compound on days 0 and 21. Monophosphorylated Lipid A (MPLA, 1 µg/animal/injection) was used as a positive control and 10% DMSO was used as vehicle control (Veh). Sera were collected on day 28 and OVA-specific immunoglobulins IgG1 (Th2 type) and IgG2c (Th1 type) were determined by ELISA (Sato-Kaneko et al., 2020). These data are presented as a scatter plot in Figure 5A that shows the distribution of compounds by their adjuvanticity profiles in inducing Th1 and Th2 responses. The data points are colored based on the type of hit (triple hits or three different dual hits) and the shape of the data point represents the adjuvanticity tier (Figure 5A, Tier 1: circles, Tier 2: squares and Tier 3: triangles). These tiers were obtained by first calculating log_10_ transformed values of the IgG1 and IgG2c titers and normalizing these values in each set for compounds between 0 and 10 (10 for MPLA and 0 for Veh). This was followed by averaging these values for IgG1 and IgG2c to obtain a combination value for each compound, where Tier 1 compounds had values > 8, Tier 2 compounds had values between 6 and 8 while Tier 3 compounds had this combination value less than 6. The dominant presence of CD63 and NF-κB hits in the Tier 1 compounds suggests the involvement of NF-κB and CD63 activation pathways for adjuvant activities. Thus, we probed the correlation between these primary screening data and the adjuvanticity to understand if these pathways involve any particular common mechanism to induce immunoglobulins. Figures 5B,C shows correlation data of primary screening data with IgG1 and IgG2c antibody titers, respectively. The analysis revealed a correlation of IgG1 titers with NF-κB and CD63 assays, but less so with IgG2c titers. Since we aimed to discover compounds that induce good adjuvanticity, we selected Tier 1 compounds (18 compounds, circles in Figure 5A) to probe into mechanisms related to immune stimulation.

### Co-Stimulatory Molecules Expression

**FIGURE 6:**
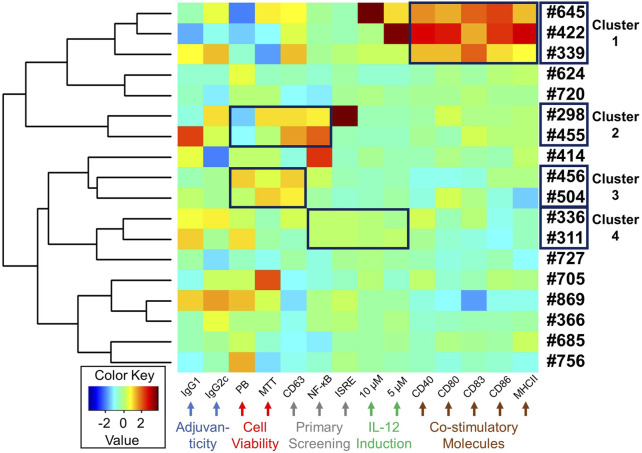
Heat map depicting a summary of biological activities of selected 18 hits. A heat map was generated for the selected 18 compounds based on all the biological data, including adjuvanticity (IgG1 and IgG2c), cell viability (MTT and PrestoBlue (PB)), primary HTS (CD63, NF-κB, and ISRE), cytokine IL-12 induction (5 and 10 µM compound concentration), and costimulatory molecules expression (CD40, CD80, CD83, CD86, and MHC class II). The absolute values from these assays were standardized and clustered for compounds presenting similar biological outcomes, as shown using a hierarchical plot on the left. This allowed us to identify 4 different clusters of compounds with similar activity profiles shown within a black box on the heat map.

Antigen presenting cells (APCs) such as dendritic cells play important roles in innate immune responses to transduce signals for subsequent humoral immunity (Liechtenstein et al., 2012). Costimulatory molecules, including CD80/86, CD40, and MHC class II molecules, are expressed on APCs and bind to their corresponding receptors on naïve or memory T cells, signalling T cell proliferation or maturation (Liechtenstein et al., 2012). While we revealed that the selected compounds had cytokine inducing effects and *in vivo* adjuvanticity when used with an antigen, the effect of these compounds on APC function was unknown. Hence, to examine whether these 18 compounds enhance maturation of immature dendritic cells, facilitating antigen-presenting cell function, mBMDCs were treated with 10 μM compound or Veh overnight and the expression of costimulatory molecules (CD40, CD80, CD83, CD86 and MHC class II) on CD11c^+^ cells was examined by flow cytometry (Figure 6, Supplementary Figure S4 and Supplementary Table S5). Of all these compounds, **#645**, **#422**, and **#339** notably enhanced the expression of costimulatory molecules (Figure 6, cluster 1).

### Heat Map Presentation of Compound Activity Profiles

Starting the HTS with 27,895 compounds, we narrowed down to 18 selected hits. We queried if there was any coherence in biological activity that would have driven the selection. Thus, we clustered the compounds by their activity profiles’ including adjuvanticity (IgG1 ang IgG2c), cell viability (PrestoBlue and MTT assays), primary HTS (CD63, NF-κB, and ISRE), IL-12 induction (10 and 5 µM compound concentration), and co-stimulatory molecules expression (CD40, CD80, CD83, CD86, and MHC class II). All these data were normalized using negative and positive controls to remove batch effects and each variable was standardized before heat maps were generated (Figure 6). Based on hierarchical clustering (shown on the left in Figure 6), we were able to group compounds into four clusters. Cluster 1 consisted of 3 compounds **#645**, **#422**, and **#339**. This group of compounds showed similar effects on the induction of co-stimulatory molecules in mBMDCs. Next, cluster 2 **(#298** and **#455**) and cluster 3 (**#456** and **#504**) consisted of compounds with similarity in their cell viability profiles as well as in the primary screenings. The last cluster 4 **(#336** and **#311**) also showed similar bioactivity in primary screenings as well as IL-12 induction.

### EV Characterization and Spider Plots

**FIGURE 7:**
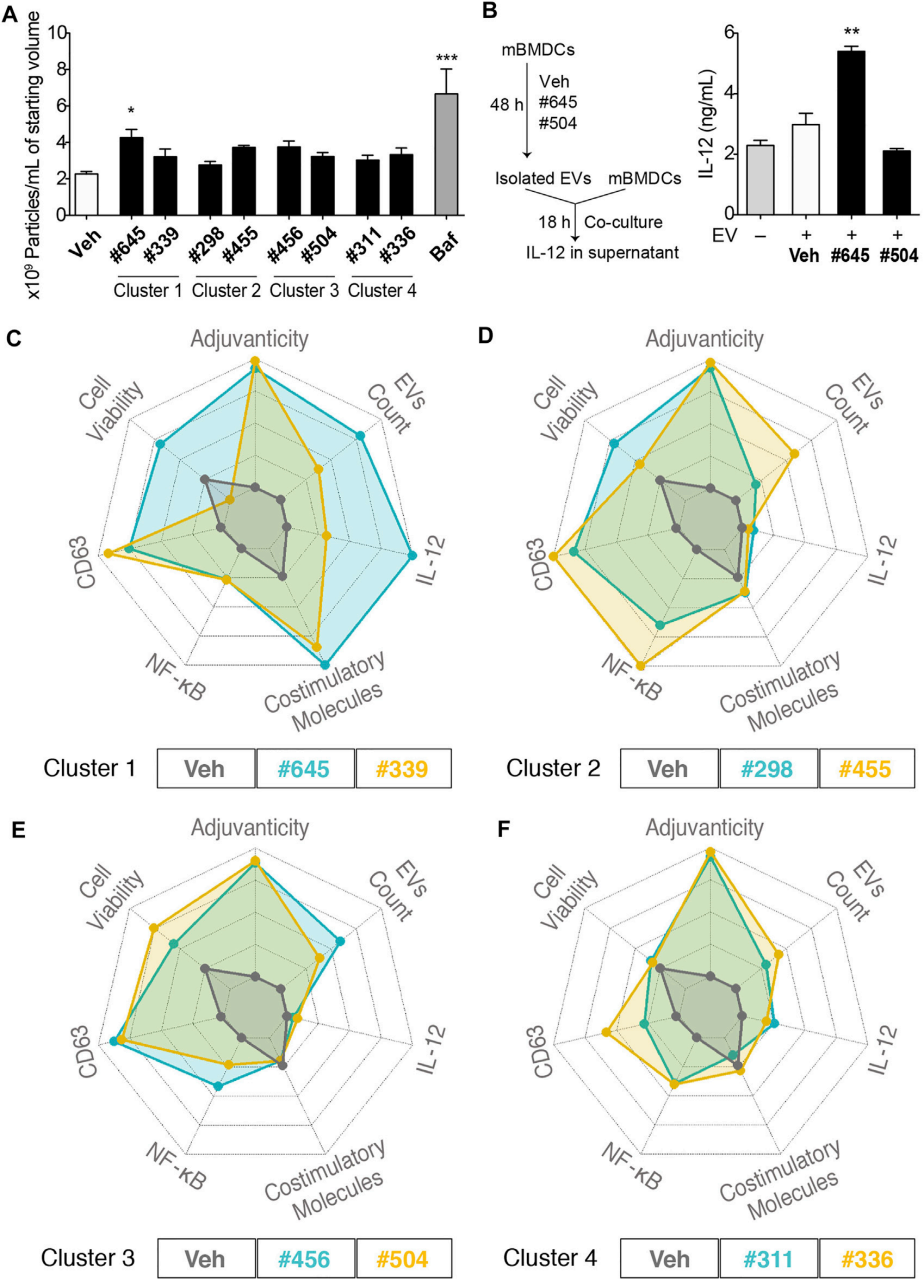
EV characterization and spider plots for selected hits depicting similar biological activities. **(A)** Number of particles per mL of starting culture medium volume assessed by MRPS using the nCS1 instruments with C-400 cartridges. mBMDCs were incubated with compound (10 µM), Veh, or bafilomycin A1 (Baf, positive control) for 48 h and EVs in the supernatant were isolated. The EVs were diluted 100-fold in 1% Tween 20-PBS and quantitated using the nCS1 system. All results were analyzed using the nCS1 Data Analyzer software. Bars indicate means ± SEM of 3-4 replicates of mBMDC batches. *p < 0.05, ***p < 0.0001, One-way ANOVA with Dunnett’s post hoc test. **(B)** Co-culture of mBMDCs with isolated EVs. mBMDCs were cultured with 10 μM **#645**, **#504** or Veh for 48 h and EVs in the culture supernatant were isolated and resuspended in 50 μL PBS. Freshly prepared mBMDCs (10^5^/100 μL) were mixed with 7 μL of the EVs and co-cultured for 18 h. IL-12 levels in the supernatant were evaluated by ELISA. Bars indicate means ± SD of duplicate wells. *p < 0.05, One-way ANOVA with Dunnett’s post hoc test vs. Veh. Spider plots show selected activity profiles’ overlay for compounds within the cluster. These activity profiles include 1. Adjuvanticity as a composite of IgG1 and IgG2c titers, 2. EVs particle concentration, 3. IL-12 induction, 4. Composite value for the expression of co-stimulatory molecules including CD40, CD80, CD83, CD86 and MHC class II, 5. NF-κB HTS data, 6. CD63 HTS data, and 7. Cell viability by MTT. The figures are shown as overlays of activity profiles of Veh with **(C)** Cluster 1 compounds **#645** and **#339**; **(D)** Cluster 2 compounds **#298** and **#455**; **(E)** Cluster 3 compounds **#456** and **#504**; **(F)** Cluster 4 compounds **#311** and **#336**. These clustered compounds show similar activity profiles’, thus validating the HTS.

To quantitate EV release from mBMDCs, we selected these clustered 8 compounds (compound **#422** had very poor aqueous solubility and was not evaluated for EV particle count) and assessed EV particle numbers in the culture supernatants of mBMDCs cultured with 10 µM compound for 48 h using microfluidic resistive pulse sensing (MRPS) with a nCS1 Instrument (Spectradyne, Signal Hill, CA). The EVs in the culture supernatants were isolated following the multistep differential ultracentrifugation protocol as previously described (Shpigelman et al., 2021). Only compound **#645** increased the number of EV particles released in the culture supernatant compared to Veh control (Figure 7A). Immunoblots of isolated EV pellets confirmed enrichment for the tetraspanins CD81 and Tsg101 (Supplementary Figures S5, S6). We also examined if the immunogenicity was from direct compound stimulation of cells or could be transferred by EVs from stimulated cells (Figure 7B). Two compounds were selected as relatively high and low inducers of EV release (**#645** and **#504**, respectively). The EVs from **#645**-treated cells stimulated higher levels of IL-12 release than those from Veh-treated cells indicating that the EVs from the cells are capable of innate immune stimulation.

To compare activity of the clustered compounds from the heat map above, their multiple bioactivities, including adjuvanticity and co-stimulatory molecules expression, were used to generate spider plot overlays along with the EV particle count data. For cluster 1, compounds **#645** and **#339** had equipotent activity in CD63 and NF-κB assays and co-stimulatory molecules expression, although they varied in IL-12 induction and cell viability, which translated as well to quantities of EVs (Figure 7C). Thus, **#645** that belongs to a 3-pyridyl-oxadiazole scaffold was revealed as a promising lead chemotype (Supplementary Figure S6). For cluster 2, compounds **#298** and **#455**, both had moderate effects on induction of cytokine IL-12 and expression of co-stimulatory molecules, but had similar CD63 and NF-κB activities in the primary HTS and both showed high values (indicating possible cell proliferation) in the MTT assays (Figure 7D). The EV particle counts inversely varied with MTT assay data suggesting distinct properties from cluster 1 compounds and pointed towards mechanisms that may involve increased formation of EVs due to decreased cell numbers (cells breaking down to release EVs). Cluster 3, consisting of compounds **#456** and **#504**, had similar activity profiles as that of cluster 2 compounds in terms of higher MTT values inversely relating to EV particle count size but had much lower NF-κB inducing activity (Figure 7E). Cluster 4, consisting of compounds **#311** and **#336**, showed very similar activity profiles, Through a rigorous tiered screening process, we discovered that these 2 compounds share structural similarity and belonged to the same 4-thieno-2-thiopyrimidine chemotype (Figure 7F and Supplementary Figure S7).

## Discussion

In immune responses, EVs have been reported to play immune-stimulatory and -suppressive roles. As a screening tool we utilized a human cell line from a lineage that has antigen presentation functions. Dendritic cells (DCs) are the most effective cell type for antigen presentation to immune cells and EVs released from DC can stimulate pro-inflammatory responses (Zitvogel et al., 1998; Escudier et al., 2005; Morse et al., 2005; Besse et al., 2016). Moreover, EVs are known to play a role in acquired immunity, as EVs released from macrophages and DCs display major histocompatibility (MHC) class Ι and ΙΙ molecules, co-stimulatory molecules (CD80 and CD86), and the adhesion protein ICAM-1 (CD54) on their surface (Admyre et al., 2006; Schorey et al., 2015; Wen et al., 2017; Lindenbergh and Stoorvogel, 2018). Antigen presentation to T cells can occur directly by MHC molecules loaded with the antigenic peptide on the surface of EVs or the EVs can be taken up by DCs or macrophages and the antigen processed to be presented by their MHC surface molecules. We developed a HTS to specifically identify chemicals that would increase biogenesis and release of EVs to enhance antigen specific immune responses in an adjuvant role. We identified **#645** exhibiting not only intrinsic immunostimulatory activity but also induce release of immunostimulatory EVs (Figures 7A,B). As **#645** enhanced the expression of MHC class II and costimulatory molecules on mBMDCs (Figure 6), the EVs isolated from **#645**-treated mBMDCs may also have these surface proteins, contributing to antigen presentation and augmenting T cell responses. Furthermore, the *in vitro* co-culture study with EVs and mBMDCs showed that the EV released from **#645**-treated mBMDCs induced IL-12 release (Figure 7F), suggesting that these EVs may also induce innate immune responses from other surface interactions like PRRs.

The use of EVs as a vaccine platform is under intensive investigation. EVs can be harvested from the supernatants of cells engineered to produce antigens and/or have specific cargos like mRNA (Kanuma et al., 2017; Anticoli et al., 2018; Jafari et al., 2020). In the first case the released EVs can preserve the native conformation of the antigenic proteins for delivery to the lymphoid system. In the second system the vaccine recipient would express the proteins at the site of delivery. The distribution of EVs administered *in vivo* is dependent on the cell source of the EVs and the route of administration (Wiklander et al., 2015). EVs administered intravenously in mice are rapidly cleared from the circulation, with a half-life of 2–4 min with complete clearance after 4 h (Takahashi et al., 2013). These EVs preferentially accumulate in the liver and spleen and are largely taken up by macrophages, which participate in the clearance of EVs (Imai et al., 2015). After half an hour EVs start to be eliminated by hepatic and renal clearance mechanisms which is completed within roughly 6 h (Takahashi et al., 2013; Lai et al., 2014). EVs that are administered subcutaneously have less hepatic uptake and a slower clearance. The long term aim of our strategy is to use a chemically controlled release of EVs at the site of antigen administration, that would enable the recipients’ cells to produce EVs continuously over time. This release is likely to be slower than a bolus of EV administration, but the continuous production would potentially overcome the limitation of rapid clearance.

Our CD63 HTS demonstrated that there are many different chemical scaffolds that induce the release of EVs, further reinforcing that the release of EVs is an important intercellular communication mechanism potentially inducing the stimulation of multiple intracellular pathways. To reduce our pool of hit candidates, we performed parallel screens on two other reporter lines, NF-κB and ISRE-*bla* reporter cells utilizing the same parent THP-1 cells. Compounds from each of the intersecting groups were able to stimulate an *in vivo* immune response to a test antigen above that of the antigen without any adjuvant. This indicates that the use of a triple screen still allowed for a broad ability to capture multiple potential leads without skewing to a single mechanism of action. Choosing Maybridge compound library for HTS enabled us to identify relatively unexplored compounds and thus we could not find any literature precedence for immunomodulatory activities by these identified hits or substructure compounds of these chemotypes using Scifinder and PubChem databases. However, our tiered screening process selected two compounds that belong to 4-thieno-2-thiopyrimidine scaffold having identical screening profiles for cytokine stimulation and cell surface marker induction thus supporting an internal reproducibility and validating our overall screening.

Although we utilized three parallel screens to identify compounds, the initial *in vivo* testing indicated that compounds with all three activities were effective adjuvants. Among the three HTS, the compounds that had the ability to stimulate EV release and NF-κB activity with or without ISRE activity were found to be the most potent adjuvants. The compounds that were the most effective that had ISRE activity also had NF-κB activity and high EV release (triple hits). In contrast, others have identified molecules that stimulate type I interferon release and ISRE activity that resulted in potent activity as adjuvants (Martínez-Gil et al., 2013). Our findings may be relative only to the compounds that were included in the library and may not be generalizable to other systems. In addition, we carefully screened compounds for *in vitro* toxicity with both PrestoBlue and MTT assays. Thus, it is not likely that NF-κB and CD63 inducing activity was due to toxicity. Although this study did not formally evaluate the safety profiles of the active compounds *in vivo*, externally isolated and administered EVs have been reported as having an acceptable safety profile including after multiple rounds of administration. (Maji et al., 2017; Zhu et al., 2017; Mendt et al., 2018; Saleh et al., 2019).

Here we have described the identification of novel chemical scaffolds that are effective *in vivo* adjuvants. Screening for the release of EVs in addition to the activation of known immunologic signaling pathways added a new additional dimension that identified robust scaffolds for further SAR studies of vaccine adjuvant activity. Chemical induction of EV release may prolong the delivery of antigen to the lymphoid system. Further studies on the induction of long-term immune memory are warranted.

## Materials and Methods

### Cell Lines

CellSensor^®^ NFκB-*bla* human monocytic leukemic THP-1 cell line was purchased from Thermo Fisher Scientific (Waltham, MA). The ISRE-*bla* THP-1 cell line was developed as described earlier (Shukla et al., 2018). These cell lines contain NF-κB and ISRE reporter constructs that uses a β-lactamase reporter gene which on activation results in beta-lactamase production and shifts the fluorescence emission of the beta-lactamase substrate [LiveBLAzerTM-FRET B/G (CCF4-AM), Thermo Fisher Scientific] to favor coumarin (460 nm emission) over fluorescein (530 nm emission).

CD63-Tluc-CD9EmGFP THP-1 reporter cells were prepared by Thermo Fisher Scientific as described previously (Shpigelman et al., 2021). Briefly, a construct with dual reporters consisting of two tetraspanins, CD63 and CD9 reporter constructs; CD63-Tluc and CD9-EmGFP, was transduced into THP-1 cells. The Tluc activities of EVs shed from CD63-Tluc-CD9EmGFP reporter cells in the culture supernatant were quantitatively measured for EV release.

All THP-1 reporter cells were maintained in 4-(2-hydroxyethyl)-1-piperazineethanesulfonic acid (HEPES) buffered RPMI 1640 medium (#72400, Thermo Fisher Scientific) supplemented with 10% dialyzed FBS (dFBS, #26400044, Thermo Fisher Scientific), 100 U/ml penicillin, 100 μg/ml streptomycin, 1 mM sodium pyruvate, 1 × MEM non-essential amino acids (NEAA), and 5 μg/ml blasticidin at 37°C in 5% CO_2_. All the HTS assay validations were carried out in assay medium OptiMEM^®^ I Reduced Serum Medium (#31985-070, Thermo Fisher Scientific) in 384-well plates (#3712, Corning).

### Reagents

The Maybridge library series, including the Maybridge HitFinder library (14,303 compounds) and the Maybridge HitCreator library (13,592 compounds) were purchased from Thermo Fisher Scientific (Leeds, United Kingdom) (Supplementary Table S1).

LPS used as a positive control for the NF-κB HTS was obtained from Sigma-Aldrich (St. Louis, MO). MPLA was purchased from Invivogen (San Diego, CA). Human IFN-α (#11101–1, PBL Assay Science) was used as a positive control for ISRE-*bla* HTS. Phorbol 12-myristate 13-acetate (PMA, BP685-1, Thermo Fisher Scientific) was used as a positive control for HTS using CD63-Tluc-CD9EmGFP reporter cells. MTT was purchased from Thermo Fisher Scientific. Ovalbumin (OVA) was obtained from Worthington Biochemical Co. (Lakewood, NJ). PBS (#14190, Thermo Fisher Scientific) filtered through a 0.02 μm inorganic membrane filter (#6809-2002, Millipore, Burlington, MA) was used to wash and dilute EVs.

### High Throughput Screens and Statistical Analysis

The robotic HTS using the three reporter cells were performed using 384-well plates by the SelectScreen™ service, Thermo Fisher Scientific (Madison, WI) (Pu et al., 2012; Chan et al., 2017b; Shukla et al., 2018). For HTS using NF-κB-*bla* and ISRE-*bla* THP-1 cells, LPS (100 ng/ml) and human IFN-α (50 nM) were used as the positive controls, respectively, while 0.5% DMSO was used as vehicle (Veh). The cells were incubated with compounds (10 µM) for 5 h, and LiveBLAzer™ FRET B/G substrate (CCF4-AM) mixture was added. Fluorescence was measured at an excitation wavelength of 405 nm, and emission wavelengths of 465 and 535 nm. The background values were subtracted from the raw values (cell-free wells at the same fluorescence wavelength). Emission ratios were calculated by dividing background-subtracted values from emission wavelength of 465 nm by those from emission wavelength of 535 nm. The response ratio (RR) was calculated as follows (emission ratio of a test well)/(average emission ratio of wells with Veh). Further, for comparison of activity, “%activation” for each compound was computed within the plate as 100 × (compound RR—average Veh RR)/(average LPS RR—average Veh RR).

In the HTS using CD63-Tluc-CD9EmGFP reporter cells (CD63 HTS), PMA (10 ng/ml) was used as a positive control (Shpigelman et al., 2021) and 0.5% DMSO was used as negative control (vehicle, Veh). Briefly, the harvested cells were resuspended in assay media containing 10% exosome-depleted FBS (#A2720801, Thermo Fisher Scientific) and plated at 2 × 10^5^ cells/mL (50 µL/well of 384-well plates) (#3674, Corning). Test compounds were added at a final concentration of 10 µM to cells and incubated for 48 h at 37°C. Subsequently, the plate was centrifuged, supernatant (25 µL) was transferred, and chemiluminescent was measured as recombinant luciferase activity (RLU) after 10 min incubation with TurboLuc assay reagent (TurboLuc™ One-Step Glow Assay kit, Thermo Fisher Scientific). The response that measures activation and subsequent release of EVs was calculated using the following formula; %response = 100 × (compound RLU—average Veh RLU)/(average PMA RLU—average Veh RLU). The viability of cells was assessed using PrestoBlue reagent (Thermo Fisher Scientific). Briefly, PrestoBlue reagent (2.5 µl) was added to the remaining cells and incubated for 30 min at room temperature, followed by fluorescence readout at (Ex 560 nm/Em 590 nm) which was normalized to fluorescence data of the Veh wells within the plate to obtain “%viability” calculated as 100 × (compound fluorescence/average Veh fluorescence).

### Data and Statistical Analysis for Selection of Hits

In each of the 3 screens, compounds were assayed twice in two replicate HTS experiments (experiments 1 and 2 performed on different days). The HTS readout was evaluated as %activation as mentioned above). For the NF-κB and ISRE HTS, hit compounds were identified using two statistical methods 1) “Top X” threshold approach and 2) “Gaussian mixture model (GMM)” approach. A compound identified by either of these methods was considered a hit. However, for the CD63 HTS, only the Top X method was used to identify hits.

Top X method: In the Top X method, all compounds with %activation values above a given threshold were selected. The threshold was computed for each plate, using the Veh wells (cells treated with 0.5% DMSO), as the mean + 3SD of %activation from these wells. Any selected compound was considered to be a false-positive if both coumarin and fluorescein values were extreme outliers according to the manufacturer’s instruction (Thermo Fisher Scientific). When a test compound was selected as a hit in both of the 2 independent HTS experiments, we reported it as a Top X hit (Figures 2A,D). For the CD63 HTS, we used the mean %response of the Veh wells as the per-plate threshold value.

GMM method: Since all compounds were assayed twice in two independent experiments, we could identify hits using a GMM. In this approach %activation data from the two independent experiments were used to construct bivariate GMM (Hastie and Tibshirani, 1996) implemented in the R-mclust package (Scrucca et al., 2016). These models were used to cluster compounds into hit or non-hit categories Briefly, the arbitrary number of 20 units was added to all %activation values to ensure all values were greater than 0 and the data were log_10_ transformed. The two independent experiments were visualized using MA (log ratio vs. average) plots, in which the *X*-axis was the average of log_10_ (%activation +20) for two replicate values for each test compound and the *Y*-axis was the difference in these values. A GMM was then fit to the plotted data, where the Bayesian Information Criterion (BIC) was used to determine the optimal number, shape and orientation of the gaussian clusters. Since this method was heavily influenced by a large number of the compounds with low %activation values, a two-step approach was employed: at the initial step, a null cluster was identified in which the majority of compounds had %activation levels similar to those from Veh-treated wells (Figures 2B,E). This null cluster was removed, as were all compounds with average activity values lower than the maximum value of this null cluster (red dotted line, Figures 2B,E). At the second step, a second GMM was fitted, using the remaining data. Apparent false-positive clusters were identified and used to construct linear boundaries, and finally compounds from the remaining clusters within these boundaries were considered GMM hits (Figures 2C,F). The GMM method ensured both a larger number of hits and also higher confirmation rate when data from an initial independent pilot screen was used to estimate the hit confirmation rate.

R statistical software (R version 3.6.1, www.r-project.org) was used for selection of hits. For the data other than HTS, Prism 6 (GraphPad Software, San Diego, CA) statistical software was used to obtain *p*-values for comparison between groups (*p* < 0.05 was considered significant) and for Spearman’s rank correlation to test for a non-zero correlation between antigen-specific antibodies and HTS data.

### Animals

Wild type C57BL/6 mice were purchased from the Jackson Laboratories. All animal experiments received prior approval by the Institutional Animal Care and Use Committee (IACUC) for UC San Diego.

### Generation of mBMDCs

mBMDCs were prepared from bone marrow cells harvested from femurs of C57BL/6 mice as previously described (Lutz et al., 1999; Datta et al., 2003). Briefly, murine bone marrow cells were harvested from C57BL/6 mice. The cells were cultured with murine granulocyte-macrophage colony-stimulating factor (GM-CSF, 20 ng/ml) for 7–8 days. Non-adherent cells were harvested and used for experiments.

### Cell Viability Assay

mBMDCs (10^5^ cells/200 µL/well) were treated with 10 and 5 µM of a test compound in 96-well plates overnight. After 18 h of drug treatment, MTT (0.5 mg/ml) was added to each well. The cells were lysed after 6–8 h incubation, and absorbance values at 570 and 650 nm were measured. PrestoBlue reagent (#A13261, Thermo Fisher Scientific) was used for cell viability assay in CD63 HTS as described earlier.

### Assessment of Cytokine Levels Using Primary Cells

mBMDCs (10^5^ cells/200 µL/well) were plated in wells of 96-well plates and treated with test compound (5 µM or 10 µM) or vehicle (0.5% DMSO) overnight. IL-12 levels in the culture supernatants were assessed by ELISA as previously described (Sato-Kaneko et al., 2021). Antibodies used in ELISA are shown in Supplementary Table S5.

### *In vivo* Assessment of Adjuvanticity of Compounds

C57BL/6 mice were intramuscularly injected with OVA (20 µg/mouse) mixed with a test compound (200 nnmol/mouse) or MPLA (1 µg/mouse) or Veh (10% DMSO) in 50 µL total volume on days 0 and 21 and bled on day 28. OVA-specific IgG1 and IgG2c levels in sera were evaluated by ELISA as described previously (Chan et al., 2009).

### Flow Cytometric Analysis for Costimulatory Molecules

mBMDCs (10^5^ cells/200 µL/well) were treated with a test compound (10 µM) or Veh (0.5% DMSO) overnight and then the costimulatory molecule expression on mBMDCs was evaluated using flow cytometry. The cells were stained with antibodies for CD11c, CD80, CD83, CD86, CD40, and MHC class II (antibodies used are listed in Supplementary Table S5). Dead cells (DAPI ^high^) were excluded from the analysis. Percent positive population of CD80, CD83, CD86, CD40, or MHC class II in the gated CD11c population were analyzed (Supplementary Figure S4).

### Heat Maps

Variables used to make a heat map were normalized and scaled by subtracting the mean and dividing by the standard deviation. Hierarchical clustering was performed, where the distance measure was the Spearman rank correlation. The R-*gplots* package was used to make heat maps.

### EV Isolation by Differential Ultracentrifugation

EVs were isolated following the protocol described in the previous study with minor modifications (Shpigelman et al., 2021). Conditioned culture media (40 ml) was spun at 300 *g* for 10 min to remove debris. Supernatants were subsequently spun at 2,000 *g* for another 10 min followed by the 10,000 *g* step for 30 min. Next, 30 ml of supernatants were transferred to 31.5 ml open-top polypropylene UC tubes (358,126, Beckman Coulter Life Sciences, CA) and spun at 100,000 g_avg_ for 3 h in an SW28 rotor (K-Factor: 2,554) by Beckman Optima XL-90 Ultracentrifuge (Beckman Coulter Life Sciences). The supernatants were then gently aspirated (leaving ∼50 µL), and pellets resuspended in 30 ml cold filtered PBS. Re-suspended pellets were then spun under the same conditions as the prior spin, followed by another round of gentle aspiration and resuspension to a final volume of 50 µL in cold filtered PBS. All centrifugation steps were performed at 4°C, and resultant samples were stored at −80°C until use. All relevant data of our experiments have been submitted to the EV-TRACK knowledgebase (EV-TRACK ID: EV220165, https://evtrack.org/index.php) (Van Deun et al., 2017).

### Evaluation of EV Concentrations Released by mBMDCs

mBMDCs (7.5 × 10^5^/ml, total 40 ml) were incubated with 10 µM test compound or vehicle (0.01% DMSO) in RPMI 1640 (#11875, Thermo Fisher Scientific) supplemented with exosome depleted FBS (#A27208, Thermo Fisher Scientific) in a T182 flask (#25–211, Genesee Scientific, San Diego, CA) for 46–48 h and EVs were isolated from culture supernatant by differential centrifugation as described above. EV particle concentrations and particle size/distribution were determined by MRPS technology with nCS1 particle analyzer utilizing C-400 cartridges (Spectradyne, Signal Hill, CA). EV samples were diluted 100-fold in 1% Tween 20-PBS and run on the nCD1 instrument. All results were analyzed using the nCS1 Data Analyzer (Spectradyne). To exclude false particle events, we applied the following peak filters: Transit time (µs) from 0 to 80, symmetry from 0.2 to 4.0, diameter (nm) from 75 to 400, signal to noise ratio (S/N) at least 10.

### Spider Plots

In a spider plot, each axis represents one of the variables to be displayed. To reduce the number of axes and make for a more interpretable visualization, selected assay readouts within the same category were combined by averaging the scaled individual variables. The final derived variables each underwent min-max normalization, i. e, x_new=(x-min)/(max-min), where min and max are minimum and maximum values of variable x, with the min (max) taken over the set of candidate compounds. The innermost net of a spider plot marks the minimum value over all the compounds, whereas the outer most net marks the maximum. R-*fmsb* package was used to make spider plots.

### Immunoblotting

mBMDCs were lysed with radioimmune precipitation assay buffer (RIPA) supplemented with protease inhibitor cocktail (Roche, Manheim, Germany) and a phosphatase inhibitor (Millipore). The total protein in the samples was quantitated by Pierce micro BCA Protein Assay Kit. Two µg of cell lysate or EVs were mixed with 4×NuPAGE sample buffer (Thermo Fisher Scientific) under reducing condition with dithiothreitol (DTT, Sigma) for Tsg101 or nonreducing condition (without DTT) for CD81. When DTT, a reducing agent, was used, samples were also denatured at 95°C for 5 min prior to loading. After fractionation on NuPAGE 4–12% Bis-Tris Gels (Thermo Fisher Scientific), samples were blotted onto Immobilon-P PVDF membranes (#IPVH00010, Sigma) and blocked for 1 h in 5% BSA-TBS-T at RT. The blots were then incubated with primary antibodies (Ab): anti-CD81 Ab (1:1,000 dilution), anti-Tsg101 Ab (1:500 dilution) overnight at 4°C with gentle agitation. After washing, the membranes were incubated with corresponding secondary antibody for 30 min at RT with gentle agitation. Blots were developed with ProSignal Dura ECL Reagent (Prometheus Protein Biology Products, Genesee Scientific, San Diego, CA) and visualized using a ChemiDoc Imaging System (Bio-Rad Laboratories, Hercules, CA). AccuRuler Prestained Protein Ladder (Lamda Biotech, St. Louis, MO) was used for the molecular weight markers. Details for antibodies and reagents are shown in Supplementary Table S5.

### Co-Culture Study With mBMDCs and Isolated EVs

mBMDCs (7.5×10^5^/ml, total 40 ml in T182 flask) were treated with 10 μM **#645**, **#504** or Veh (0.01% DMSO) in RPMI 1640 (Thermo Fisher Scientific) supplemented with exosome depleted FBS (10%, Thermo Fisher Scientific) for 46–48 h. The EVs were isolated from the conditioned media and resuspended in 50 μL filtered PBS at the final step as described above. Freshly prepared mBMDCs (10^5^ cells/100 μL) were mixed with 7 μL of the EVs in a well of 96-well plate and incubated for 18 h. IL-12 levels in the culture supernatants were assessed by ELISA.

## Data Availability

The raw data supporting the conclusion of this article will be made available by the authors, without undue reservation.
