# Supplementary material for: A Triple High Throughput Screening for Extracellular Vesicle Inducing Agents With Immunostimulatory Activity
Source: Front Pharmacol. 2022 Apr 11;13:869649. doi: 10.3389/fphar.2022.869649 (PMC9035538; doi:10.3389/fphar.2022.869649)
Supplement: Supplementary file 1 [file DataSheet1.PDF]

Supplementary Table S1 – Number of compounds from MayBridge library used in HTS

	HitFinder	HitCreator	Total
# of compounds	14400	14000	28400
Compounds removed due to UK restrictions[(a)]	94	190	284
Compounds damaged[(b)]	3	218	221
Compounds in pilot screen	1035	1176	2211
Compounds in HTS screen[(c)]	14303	13592	**27895**

(a) Due to export restrictions in UK, 287 compounds were not shipped.
(b) An additional 221 compounds were removed due to damage during the shipping.
(c) A total of 27895 compounds were screened in the HTS.

Supplementary Table S2 – Three reporter cell lines used in the HTS.

Reporter cell lines	Purpose to measure	Detection method
CD63Tluc-CD9EmGFP THP-1 reporter cells	EV release	Turboluciferase activity
CellSensor™ NF-κB-*bla* THP-1 cell line	NF-κB activation	Beta-lactamase FRET activity
ISRE-*bla* THP-1 cell line	ISRE activation	Beta-lactamase FRET activity

Supplementary Table S3 – Number of hits by hit identification methods

HTS	Top X hits[(a)]	GMM hits[(b)]	Common[(c)]	Total hits[(d)]
NF-κB HTS	398	497	319	576
ISRE HTS	481	444	383	542
CD63 HTS	12,954	--	--	12,954

(a) Number of hits identified by Top X method.
(b) Number of hits identified by Gaussian mixture model (GMM) method.
(c) Number of hits common to both Top X and GMM method of hit identification.
(d) Final number of unique hits.

Supplementary Table S4 – Number of hits by cytotoxic profile using PrestoBlue viability data

Hit Type	PrestoBlue Viability cutoff[(a)]	All Hits	>20%[(b)]	>40%[(b,c)]	>60%[(b)]	>80%[(b)]	>100%[(b)]
**Triple Hits**	**All 3 assays**	**161**	**147**	**138**	**123**	**95**	**60**
**Dual Hits**	**CD63 & NF-κB**	**296**	**280**	**254**	**223**	**173**	**90**
**Dual Hits**	**CD63 & ISRE**	**231**	**224**	**217**	**200**	**173**	**109**
**Dual Hits**	**NF-κB & ISRE**	**37**	**35**	**35**	**35**	**34**	**26**
Single Hits	CD63 only	12266	12082	11898	11574	10782	8285
Single Hits	NF-κB only	82	72	71	70	66	47
Single Hits	ISRE only	112	93	92	90	85	64
	**Total Dual and Triple Hits**	**725**	**686**	**644**	**581**	**475**	**285**

(a) Cell viability data obtained from PrestoBlue assay in THP-1 CD63 Turbo-luc reporter cells was utilized to categorized these compounds by graded cell viability data.

(b) Number of compounds above the indicated %viability value were categorized into triple, dual or single hits candidates.

(c) Compounds marked by black box which were either triple hits or dual hits and had more than 40% viability were selected as HTS hits for further biological evaluation.

Supplementary Table S5 – List of antibodies and reagents used in the study, their source and catalog numbers

	**Source**	**Cat#**
**Antibodies for ELISA**		
Purified rat anti-mouse IL-12 p40/p70	BD Biosciences	551219
Biotin rat anti-mouse IL-12 p40/p70	BD Biosciences	554476
**Antibodies (clone) for flow cytometry**		
Anti-CD80, FITC (16-10A1)	BioLegend	104706
Anti-CD86, APC/Cy7 (GL1)	BioLegend	105030
Anti-CD83, PE (Michel-17)	eBioscience	12-0831
Anti-CD11c, APC (N418)	eBioscience	17-0114
Anti-MHC II (I-A/I-E), FITC (M5/114.15.2)	eBioscience	11-5321
Anti-CD40, PE (1C10)	eBioscience	12-0401
**Antibodies (clone) for immunoblot**		
CD81 (D5O2Q) Rabbit mAb (Mouse Specific)	Cell Signaling Technology	10037
Tsg101 Monoclonal Antibody (4A10)	Thermo Fisher Scientific	MA1-23296
Anti-mouse IgG, HRP-linked Antibody	Cell Signaling Technology	7076S
Anti-rabbit IgG, HRP-linked Antibody	Cell Signaling Technology	7074S
4×NuPAGE LDS sample buffer	Thermo Fisher Scientific	NP0007
Dithiothreitol (DTT)	Sigma	D9779-5G
AccuRuler Prestained Protein Ladder	Lamda Biotech	G02101
ProSignal Dura ECL Reagent	Prometheus Protein Biology Products	20-301B

Supplementary Figure S1 – Hit confirmation rates for HTS screens compared to pilot screens

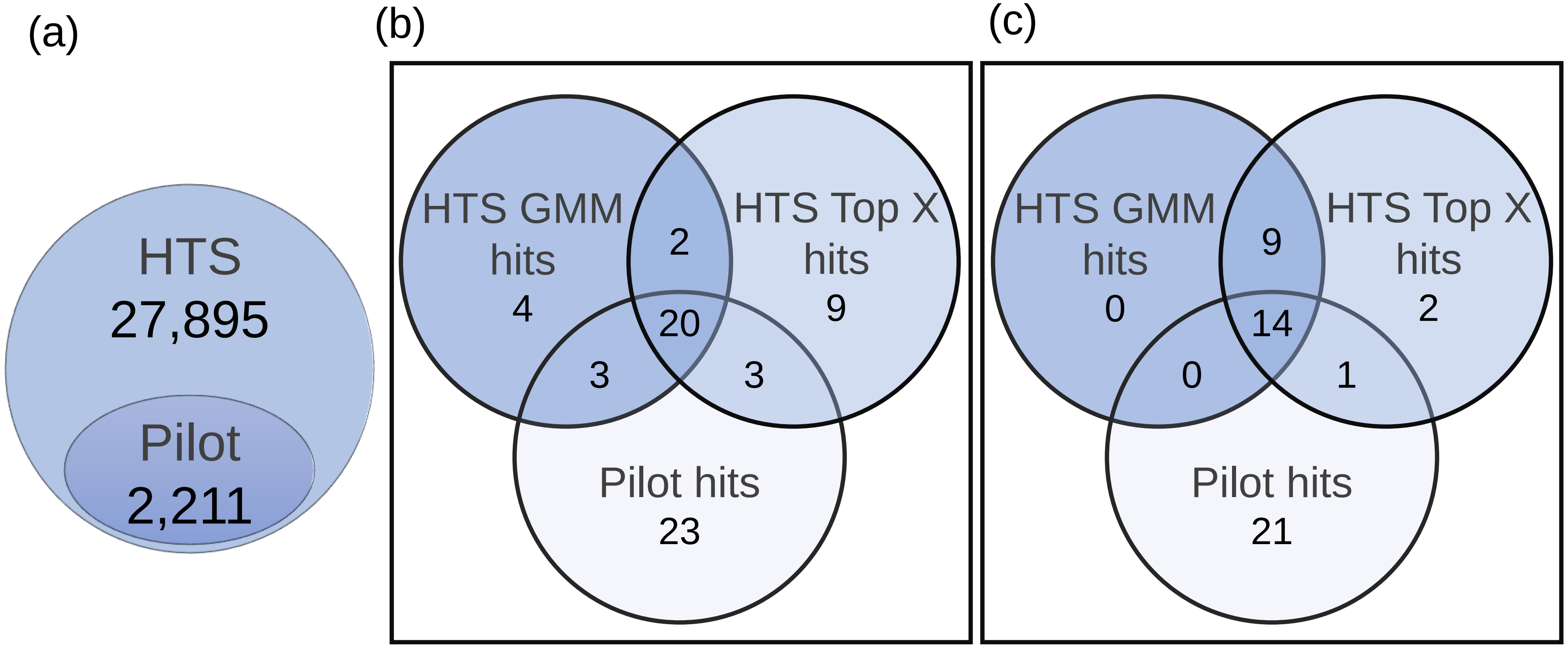

(d)

HTS	HTS confirmation rate TOP X	HTS confirmation rate GMM
NF-κB	23/34 (67.6%)	23/29 (79.3%)
ISRE	15/26 (57.7%)	14/23 (60.9%)
CD63	371/705 (52.6%)	--

(a) A graphic showing all the 2,211 compounds evaluated for pilot screens were part of the HTS screens consisting of 27,895 compounds.
(b) A Venn diagram showing number of hits identified in NF-κB HTS by GMM and Top X methods and in the NF-κB pilot screen.
(c) A Venn diagram showing number of hits identified in ISRE HTS by GMM and Top X methods and in the ISRE pilot screen.
(d) Table showing number of HTS hits common to pilot screen hits divided by number of total HTS hits and calculated confirmation rates for the Top X and GMM methods in each HTS.

Supplementary Figure S2 – Scatter plot of cytokine responses by selected compounds at two different concentrations

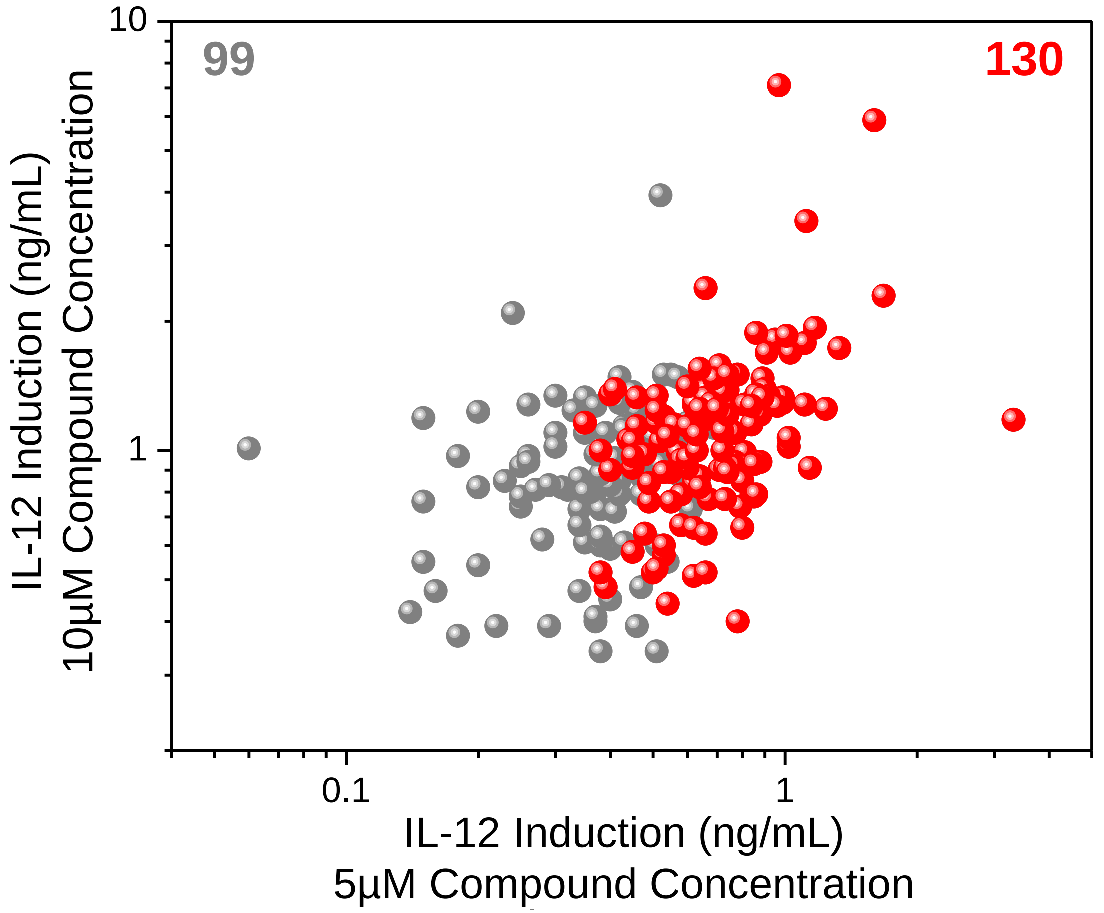

mBMDC were incubated with selected hits (X axis- 5 µM and Y axis - 10 µM compound concentrations) overnight and IL-12 release in the culture supernatant was measured by ELISA. The number shown inside the graph represent number of the selected hit candidates in red and number of excluded compounds in gray.

Supplementary Figure S3 – Structures of examples of the hit candidates eliminated by medicinal chemistry approach.[a]

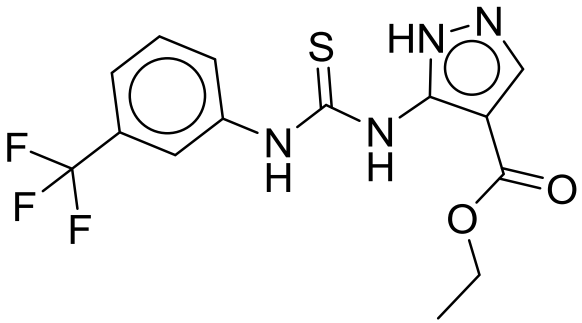

(#304)
Thiourea Liability

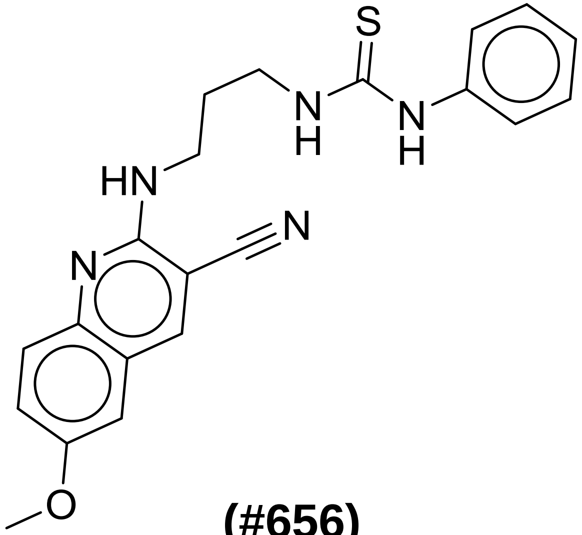

(#656)
Thiourea Liability

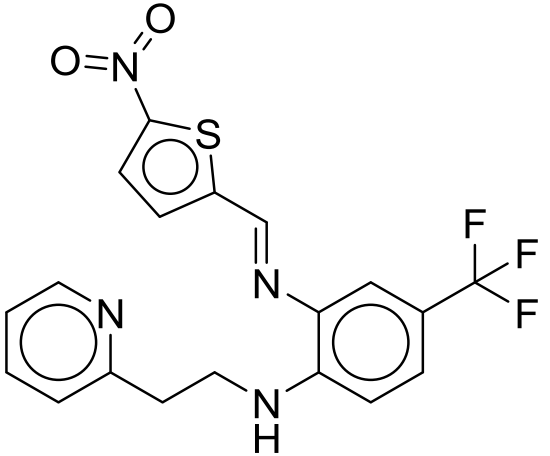

(#795)
Hydrolyzable Imine

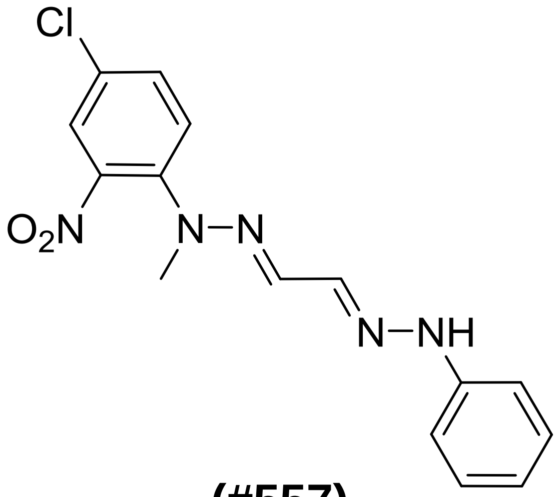

(#557)
Hydrolyzable Hydrazones

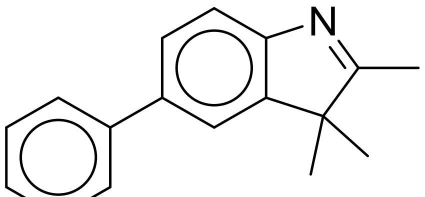

(#867)
Lack of functionality

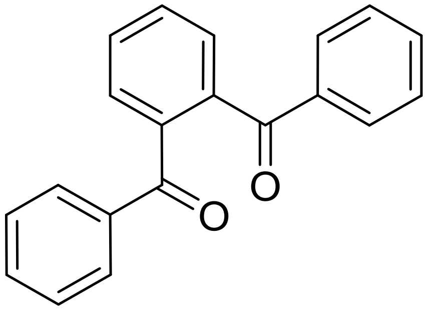

(#848)
Lack of functionality

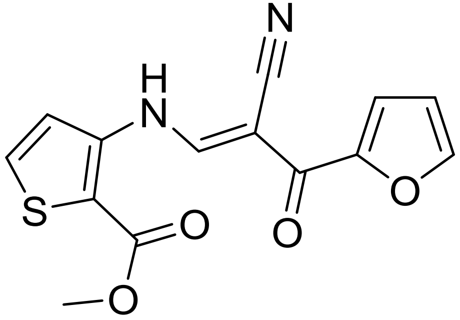

(#559)
Michael Acceptor

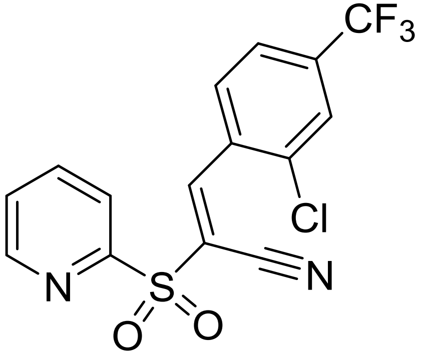

(#757)
Michael Acceptor

[a]Compound number from the HTS is shown in brackets while the chemical functionality identified for elimination of compound from the list of hits is mentioned below the structure

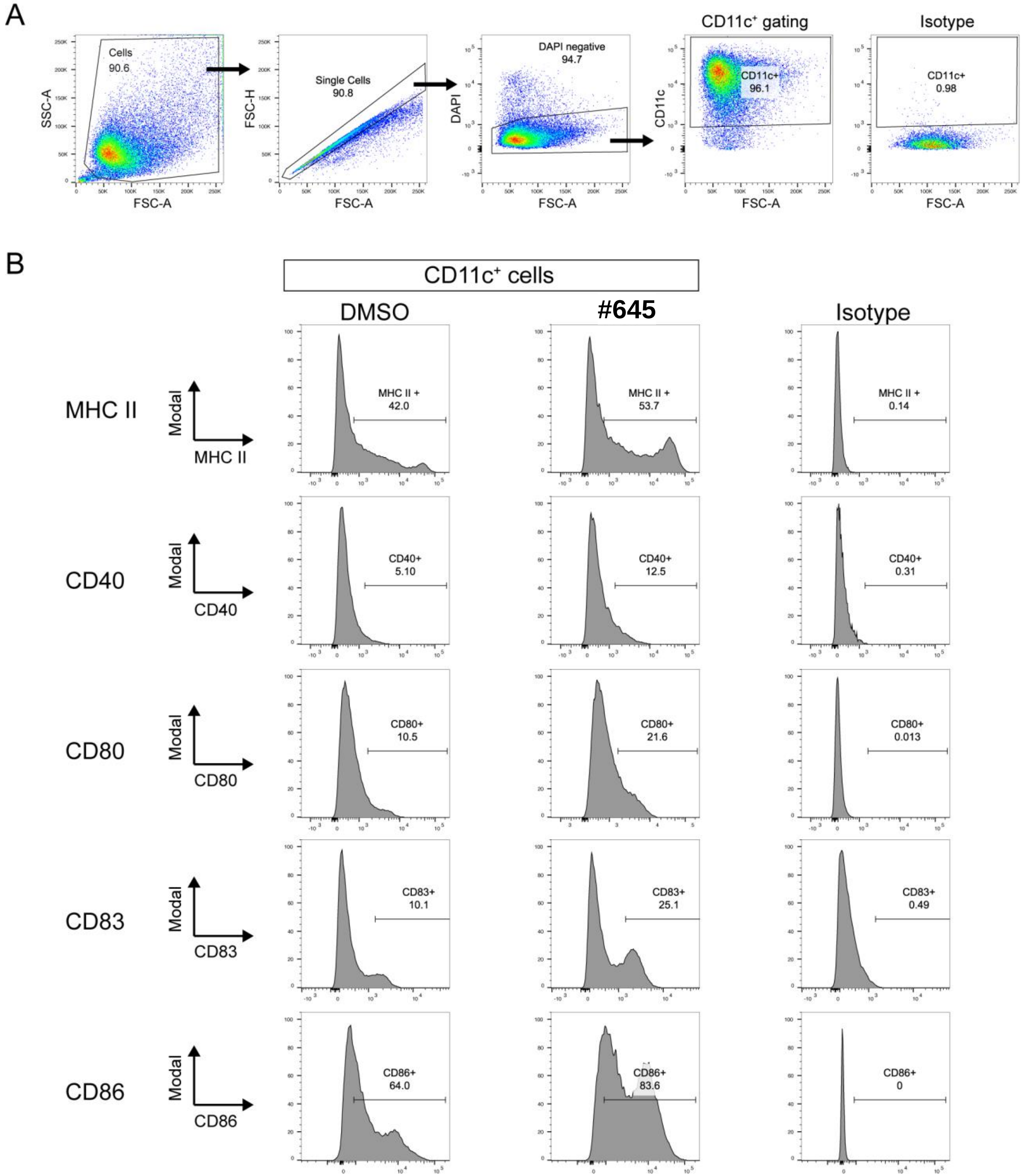

Supplementary Figure S4 – Flow cytometric analysis for costimulatory molecules on BMDCs.

BMDCs were cultured with 10 μM of compound overnight and then the cells were stained with antibodies for CD11c, CD40, CD80, CD83, CD86 and MHC class II. Dead cells (DAPI^{high}) were excluded from the analysis. The expression of costimulatory molecules on CD11c^{+} cells was examined by flow cytometry. (A) Gating strategy for CD11c^{+} cells. (B) Representative histograms for DMSO (Veh) and a representative compound #**645**. %CD40^{+}, %CD80^{+}, %CD83^{+}, %CD86^{+}, and %MHC II subsets in CD11c^{+} cells are shown. The data are normalized to Veh and were used for the heatmap and the spider plots.

Supplementary Figure S5 – EV characterization by MRPS and immunoblot

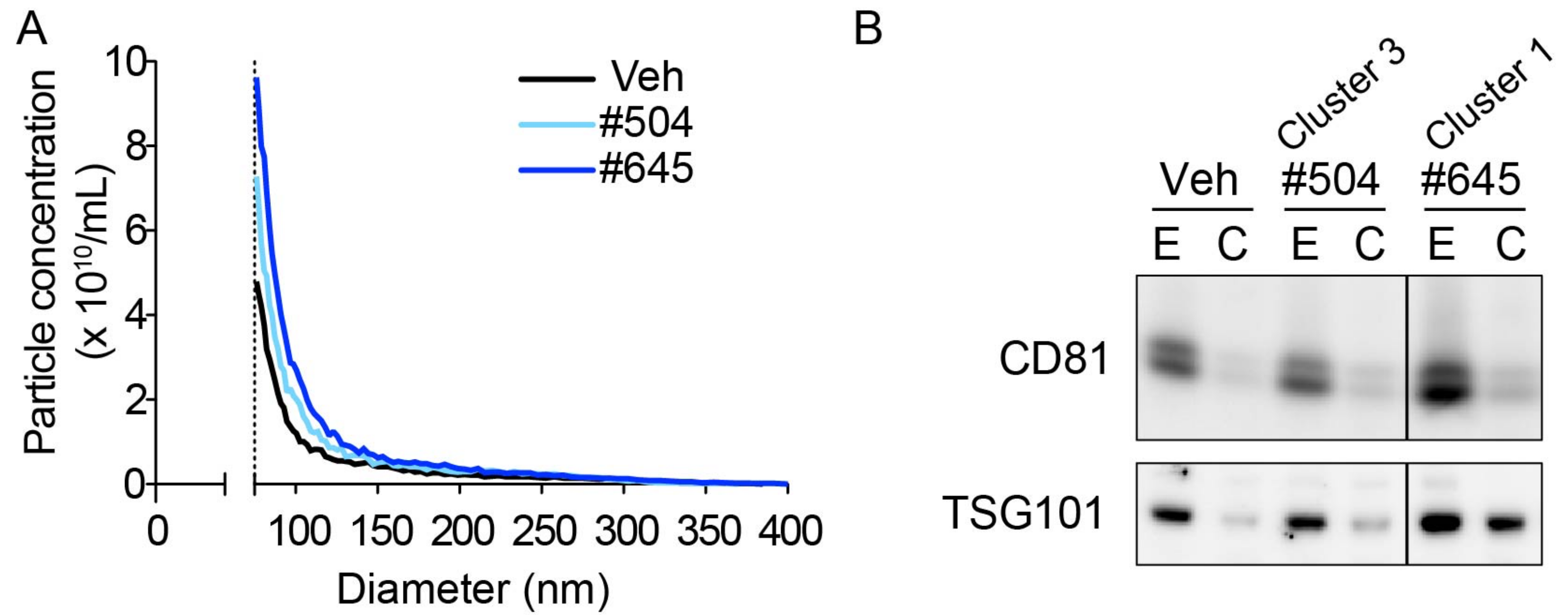

(**A**) MRPS analyses of EV samples using the instrument CSn1 (Spectradyne). Size range/distribution of EV released following incubation with representative compounds (#**645** and #**504**) and Veh (0.01% DMSO) are shown. EV samples were prepared by differential ultracentrifugation methods. Isolated EVs were diluted 100-fold in 1% Tween 20-PBS. All results were analyzed using the nCS1 Data Analyzer (Spectradyne). To exclude false particle events, the peak filters were applied: Transit time (µs) from 0 to 80, Symmetry from 0.2 to 4.0, Diameter (nm) from 75 to 400, Signal to noise ratio (S/N) at least 10. (**B**) Immunoblot of EVs (E) and parental cell lysate (C). mBMDCs were treated with #**504** (10 µM), #**645** (10 µM) or Veh. Blots were probed with anti-CD81 and Tsg101. The images shown are representative of two independent experiments. The original blots were shown in Supplementary Figure S6.

Supplementary Figure S6 – Immunoblot scans used in Supplementary Figure S5[a]

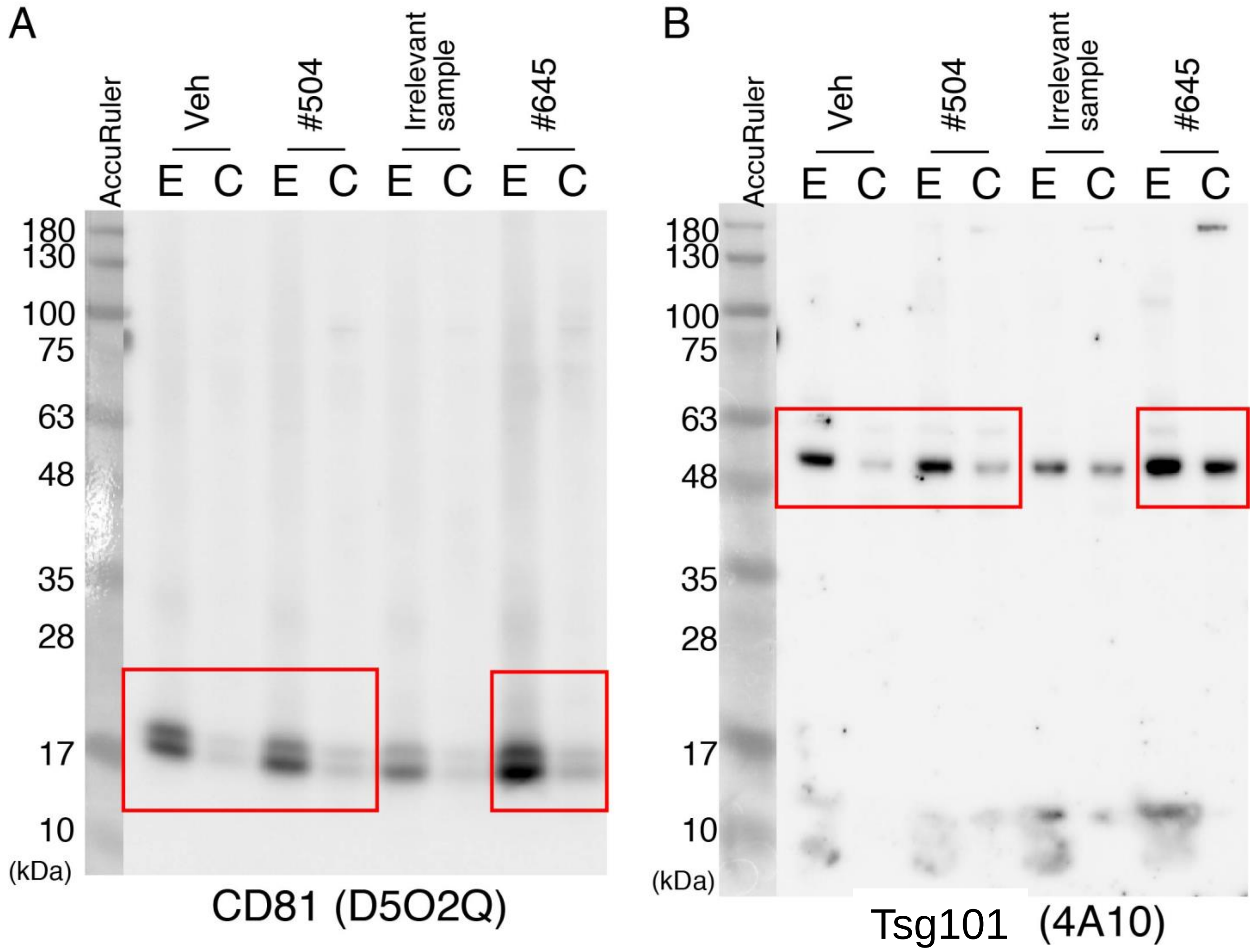

[a]The blots were probed with anti-CD81 (1:1000 dilution, **A**), anti-Tsg101 (1:500 dilution, **B**). EVs samples were run under or non-reducing and reducing conditions for CD81 and Tsg101, respectively. The molecular weight markers AccuRuler Prestained Protein Ladder (Lamda Biotech) were used. The red-dotted box indicates the bands shown in Supplementary Figure S5. Details for methods and antibodies are shown in the method section and Supplementary Table S5.

Supplementary Figure S7 – Hit chemotype scaffolds identified via HTS.[a]

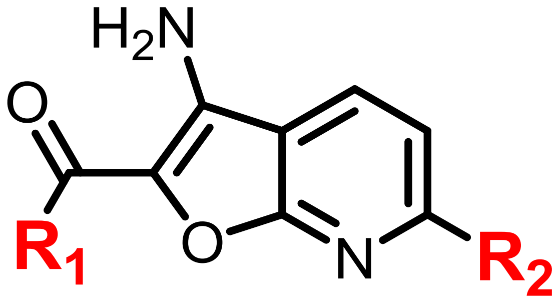

(#298)
3-Amino furopyridines

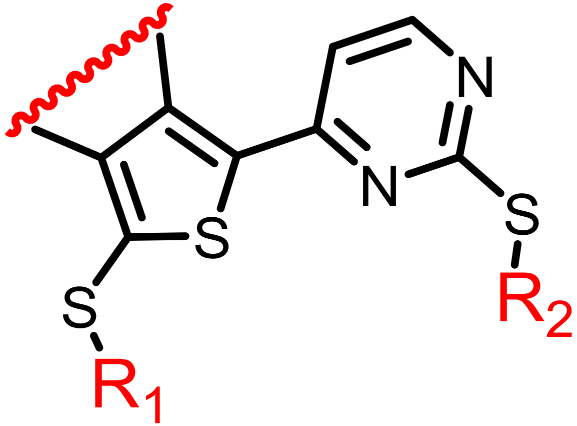

(#311, #336)
4-Thieno-2-thiopyrimidines

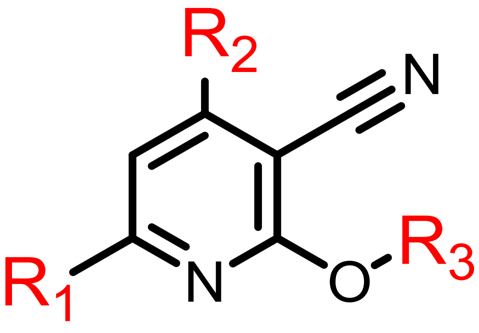

(#339)
Nicotinonitriles

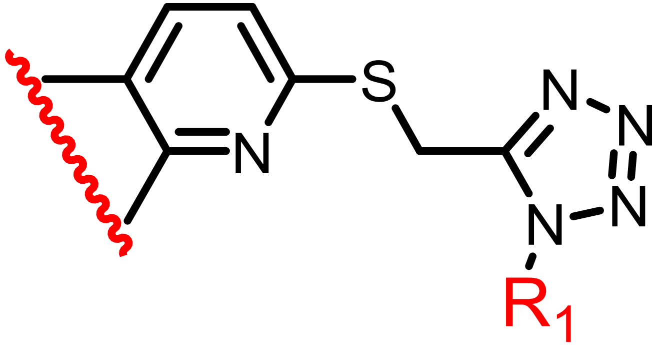

(#455)
2-S-methyltetrazolyl pyridines

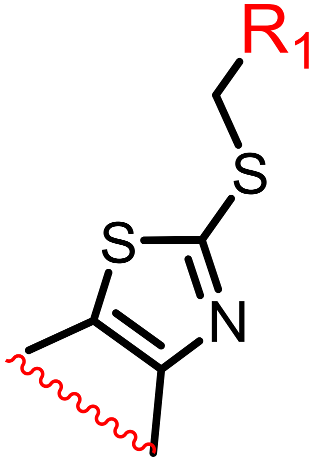

(#456)
S-alkyl thiazoles

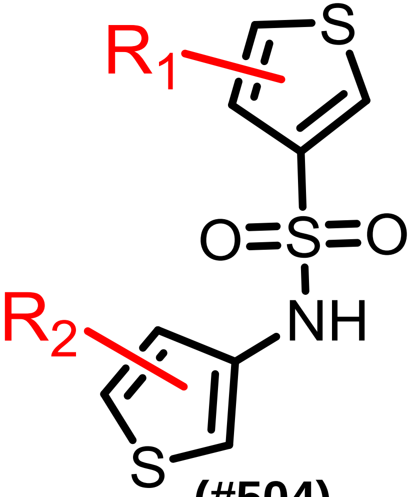

(#504)
Bisthienyl sulfonamides

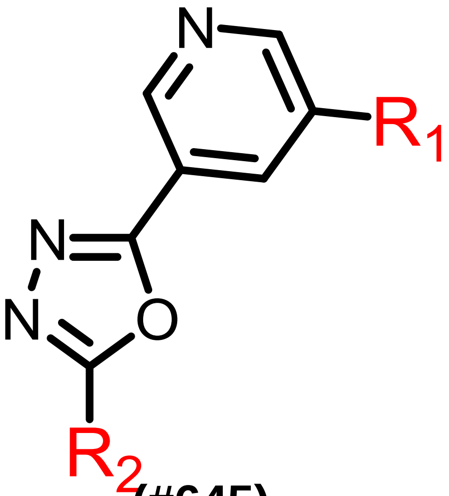

(#645)
3-Pyridyl-oxadiazoles

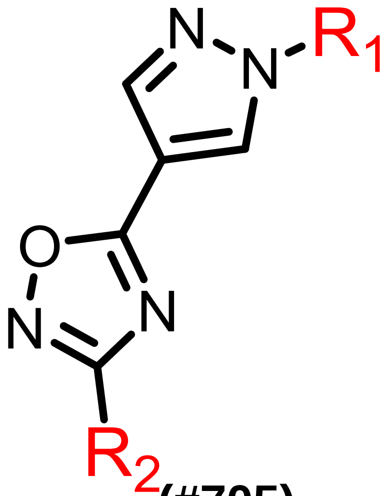

(#705)
4-Pyrazolyl-oxadiazole

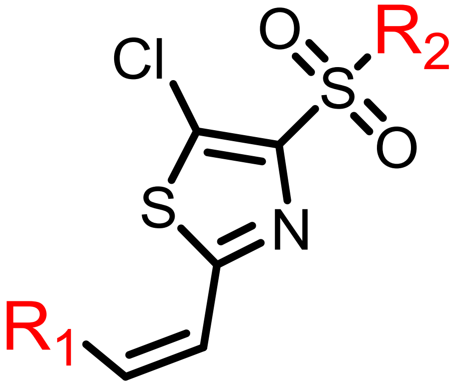

(#869)
2-Alkene-4-sulfone thiazoles

[a]Compound number from the HTS is shown in brackets while the chemical scaffold name for the compound is mentioned below the structure
